# Acute Kidney Injury in Trauma Patients Admitted to Critical Care: Development and Validation of a Diagnostic Prediction Model

**DOI:** 10.1038/s41598-018-21929-2

**Published:** 2018-02-26

**Authors:** Ryan W. Haines, Shih-Pin Lin, Russell Hewson, Christopher J. Kirwan, Hew D. Torrance, Michael J. O’Dwyer, Anita West, Karim Brohi, Rupert M. Pearse, Parjam Zolfaghari, John R. Prowle

**Affiliations:** 1 Adult Critical Care Unit, The Royal London Hospital, Barts Health NHS Trust, Whitechapel Road, London, E1 1BB UK; 2 William Harvey Research Institute, Queen Mary University of London, London, UK; 3 Department of Anesthesiology, Taipei Veterans General Hospital and School of Medicine, National Yang-Ming University, Taipei, Taiwan; 4 Division of Biostatistics, Graduate Institute of Epidemiology and Preventive Medicine, College of Public Health, National Taiwan University, Taipei, Taiwan; 5 Centre for Trauma Sciences, Blizard Institute, Queen Mary University of London, London, UK; 6 Department of Renal Medicine and Transplantation, The Royal London Hospital, Barts Health NHS Trust, Whitechapel Road, London, E1 1BB UK

## Abstract

Acute Kidney Injury (AKI) complicating major trauma is associated with increased mortality and morbidity. Traumatic AKI has specific risk factors and predictable time-course facilitating diagnostic modelling. In a single centre, retrospective observational study we developed risk prediction models for AKI after trauma based on data around intensive care admission. Models predicting AKI were developed using data from 830 patients, using data reduction followed by logistic regression, and were independently validated in a further 564 patients. AKI occurred in 163/830 (19.6%) with 42 (5.1%) receiving renal replacement therapy (RRT). First serum creatinine and phosphate, units of blood transfused in first 24 h, age and Charlson score discriminated need for RRT and AKI early after trauma. For RRT c-statistics were good to excellent: development: 0.92 (0.88–0.96), validation: 0.91 (0.86–0.97). Modelling AKI stage 2–3, c-statistics were also good, development: 0.81 (0.75–0.88) and validation: 0.83 (0.74–0.92). The model predicting AKI stage 1–3 performed moderately, development: c-statistic 0.77 (0.72–0.81), validation: 0.70 (0.64–0.77). Despite good discrimination of need for RRT, positive predictive values (PPV) at the optimal cut-off were only 23.0% (13.7–42.7) in development. However, PPV for the alternative endpoint of RRT and/or death improved to 41.2% (34.8–48.1) highlighting death as a clinically relevant endpoint to RRT.

## Introduction

Acute Kidney Injury (AKI) is a common complication of severe trauma that is independently associated with increased morbidity and mortality in patients admitted to the intensive care unit (ICU)^1,2^. As increasing numbers of trauma patients are surviving their initial life threatening illness, earlier diagnosis and treatment of complications such as AKI are important areas for intervention, aiming to improve short and long-term outcomes^3,4^. The current management of AKI in the general critical care population is early identification of AKI, limiting ongoing or recurrent renal injury and providing supportive management of advanced renal dysfunction^5^. However, traditional markers of AKI, urine output and serum creatinine, are late and non-specific biomarkers of renal dysfunction, which may preclude early preventative intervention. Consequently, there is considerable interest in strategies that target interventions in patients with evolving AKI identified using novel methodologies including risk prediction models and/or AKI-specific biomarkers^6–8^.

Major trauma patients are exposed to a multitude of risk factors for AKI, including systemic inflammation, hypovolemic shock, massive transfusion, rhabdomyolysis, abdominal compartment syndrome and major surgery^9,10^. As in trauma presentation is usually early, rapid predictive modelling for development of AKI or need for renal support might enable interventions to improve outcomes such as the early commencement of RRT. Previously described demographic risk-factors for AKI in trauma populations, include older age, greater comorbid disease and diabetes^1,2,11,12^. However, most previous studies have not integrated early biochemical and clinical data in attempting to predict AKI outcomes after major trauma.

We hypothesised that demographic, clinical, biochemical and physiological parameters around trauma-ICU admission might accurately predict the development of AKI and need for RRT in the ICU. To address this question, we performed a retrospective cohort study of trauma ICU admissions with multivariable modelling for AKI outcomes utilising distinct development and validation patient cohorts.

## Methods

We conducted a single centre, retrospective observational cohort study of trauma admissions to the Royal London Hospital Adult Critical Care Unit, a Level-1 trauma centre in central London. This study was approved by the Barts Health/Queen Mary University of London Joint Research Office as a retrospective review of data collected as part of usual patient care without requirement for research ethics committee review. We followed the TRIPOD statement guidelines for methodology and reporting of multivariable predictive modelling.

The development cohort comprised all admissions from February 1^st^, 2012 to October 31^st^, 2014 with the validation cohort admitted from November 1^st^, 2014 to May 1^st^, 2016. We considered all trauma admissions to the emergency department (ED) of the Royal London Hospital that were admitted either directly to the adult ICU or via the operating theatre. To examine a population at risk of new AKI diagnosis in hospital we excluded advanced renal dysfunction at hospital admission (first creatinine value >354 µmol/L or history of end stage renal disease) deaths within 24 h from ICU admission. As well as developing and validating predictive models for AKI outcomes, we examined the relationship between AKI of varying severity with survival and hospital length stay.

Clinical, laboratory and demographic data was collated from the following sources: The Royal London Hospital trauma admission database (Collector, Digital Innovation Inc, Forest Hill, MD, USA), the Adult Critical Care Unit intensive care national audit centre (ICNARC) database and Barts Health Cerner Millennium powerInsight data warehouse (Cerner Inc, Kansas City, MI, USA) for pathology data. Data linkage was performed by members of the clinical research team and collated records were pseudo-anonymised prior to detailed analysis. The Royal London Hospital trauma database inclusion criteria are the same as those of the national Trauma Audit and Research Network that collects information on all presenting major trauma patients (those with a hospital length of stay of 72 hours or more, and/or requiring high dependency care, and/or where a death occurred in hospital)^13^. From this larger trauma dataset, we only included patients admitted to the ICU either directly from the ED or via the operating theatre.

The primary outcome was prediction of development of AKI requiring RRT. Secondary outcomes included; prediction of all AKI (stages 1–3) and moderate-severe AKI (AKI stages 2–3) and within all AKI groups the prediction of AKI and/or death. AKI was diagnosed and staged using the serum creatinine (SCr) criteria of the 2012 KDIGO AKI guidelines; namely, any increase in SCr ≥26.5 µmol/L within 48 hours or an increase in SCr ≥1.5 times baseline SCr within 7 days^14^. AKI stage 2 was then defined as a ≥2 but <3 fold increase in SCr from peak to baseline and AKI stage 3 as a ≥3 fold increase in SCr, a rise of ≥26.5 µmol/L to ≥354 µmol/L or any AKI treated with RRT^14^. Due to the acute nature of trauma admissions baseline SCr was defined as the first documented SCr in hospital.

### Statistical analysis

Statistical analysis was performed in R v3.3.3 (R Foundation for Statistical Computing, Vienna, Austria) using RStudio v1.0.136 (RStudio Inc, Boston, MA, USA). Continuous data are presented median with interquartile range (IQR) or range with the Wilcoxon rank sum test, categorical data were compared using Fisher’s test. Cumulative incidence of the competing endpoints of death in hospital or hospital discharge alive were plotted for AKI/No AKI and more severe AKI (stage 2–3). The effect of AKI severity on hospital survival was modelled using logistic regression with adjustment for variables expected to be associated with risk of death: simplified acute physiology score-2 (SAPS-2) in the first 24 hours after ICU admission (excluding serum urea component), admission trauma new injury severity score (NISS), age and the presence of severe brain injury (an abbreviated injury score of >3 for the Abbreviated Injury Scale head component)^15^.

In predictive modelling for AKI and RRT we considered the following variables, chosen based on data availability, physiological plausibility and the existing literature: age, sex, NISS, ED systolic blood pressure, Charlson Comorbidity Index (based on ICD-10 coding from the current and any previous admissions using the mapping of Quan)^16^, units of packed red blood cells (PRBC) transfused in the first 24 h from hospital presentation and first blood results in hospital for the following assays: activated partial thromboplastin time (APTT) ratio, albumin, alanine aminotransferase (ALT), amylase, calcium, C-reactive protein (CRP), creatine kinase (CK), SCr, haemoglobin, international normalised ratio (INR), arterial blood lactate, red cell distribution width, phosphate, platelet count, total bilirubin and white blood cell count. For model building missing values were handled by simultaneous transformation and single imputation^17^. To avoid over-fitting, we undertook stepwise data-reduction using hierarchical clustering principal component analysis to define a limited set of candidate variables for model-building. For clusters containing >1 variable the first principal component of the transformed variables within the cluster was then extracted and analysed together with age and single variable clusters in logistic regression for prediction of AKI outcomes. This was followed by backward variable selection based on minimisation of the AIC. The final models for prediction of any AKI, AKI stage 2–3 and need for RRT in ICU were then selected by a further process of backward elimination. Global goodness of fit was assessed by unweighted sum of squares test and by plotting of bootstrap calibration curves. The validation dataset was assessed against the development models with no additional variable selection or model fitting. Model performance was assessed by the c-statistic in the development and validation datasets as well as diagnostic discrimination at cut-off values defined by the Youden-Index. Finally, to explore differing cut-offs and the relative contribution of predictors in determining risk we examined the variables identified in logistic regression in classification and regression tree (CART) modelling.

## Results

**Figure 1:**
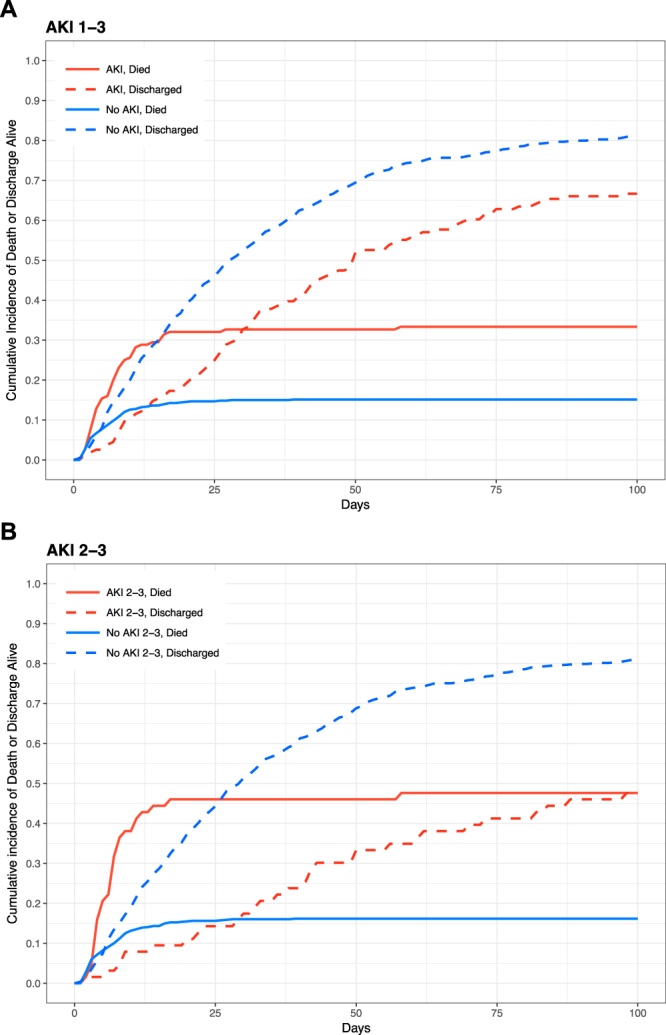
Cumulative incidence of death or discharge alive for any acute kidney injury (**A**) and acute kidney injury stage 2–3 (**B**).

After exclusions, we identified 830 patients in our development dataset (Fig. S1) of which 163 (19.6%) developed KDIGO-creatinine AKI stage 1–3 in the seven days after trauma (maximum stage 1–100, 2–13 and 3–50). Median time to maximum SCr was 1.6 days (IQR 0.8–2.6) and 2.7 days (2.3–3.7) for AKI stage 1 and AKI stage 2–3 respectively. RRT, exclusively delivered as continuous RRT, was required for 42 (5.1%) patients and median time of commencing RRT was 1 day after ICU admission (IQR 1–3). Median age was 40 years, ISS 25 (17–33), NISS 35 (24–50); 156 patients died in hospital (18.8%) – Table 1. Patients with AKI had a significantly higher hospital mortality than those without AKI (53/163, 32.5% vs. 103/667, 15.4%) and longer length of stay (Fig. 1). AKI patients were significantly older, more likely to have sustained abdominal or pelvic injury and had higher ICU illness severity scores in the first 24 h (Table 1). After adjustment for age, NISS, SAPS-2 and presence of brain injury, moderate-severe AKI (stages 2–3) was associated with increased hospital mortality (OR 5.35 95% CI: 2.73–10.47) while AKI stage 1 was non-significant (Fig. S2).

**Table 1:** Baseline characteristics, biochemical parameters and clinical outcome for patients divided into non-acute kidney injury and acute kidney injury groups.

	All	No AKI	AKI	p-value	Missing values (n)
n (%)	830 (100)	667 (80.4)	163 (19.6)		
Sex = male (%)	676 (81.4)	539 (80.8)	137 (84.0)	0.4	
Age (median [IQR])	42 [27, 57]	40 [27, 54]	50 [31.50, 66]	<0.001	
Ethnicity (%)					
White	620 (74.6)	508 (76.2)	112 (68.7)		
Black	85 (10.2)	63 (9.4)	22 (13.5)		
Asian	103 (12.4)	80 (12.0)	23 (14.1)		
Mixed race/other	21 (2.5)	15 (2.2)	6 (3.7)		
Unknown	1 (0.1)	1 (0.1)	0 (0.0)		
Charlson Comorbidty Index (%)				<0.001	
0	635 (76.5)	540 (81.0)	95 (58.3)		
1–2	159 (19.2)	106 (15.9)	53 (32.5)		
≥3	36 (4.3)	21 (3.1)	15 (9.2)		
SAPS-2 (median [IQR])	35 [28, 43]	35 [27, 41]	41.50 [33, 49.25]	<0.001	20
APACHE II (median [IQR])	12 [8, 16]	11 [8, 15]	15 [12, 19]	<0.001	19
NISS (median [IQR])	34 [24, 50]	34 [22, 50]	34 [25, 50]	0.655	11
ISS (median [IQR])	25 [17, 33]	25 [17, 33]	25 [19, 34]	0.098	11
Site of Injury					
Brain (%)	406 (48.9)	354 (53.1)	52 (31.9)	<0.001	
Chest (%)	200 (24.1)	158 (23.7)	42 (25.8)	0.65	
Abdomen (%)	84 (10.1)	52 (7.8)	32 (19.6)	<0.001	
Pelvis (%)	78 (9.4)	48 (7.2)	30 (18.4)	<0.001	
Limbs (%)	68 (8.2)	52 (7.8)	16 (9.8)	0.498	
Spine (%)	105 (12.7)	89 (13.3)	16 (9.8)	0.279	
Face and Neck (%)	67 (8.1)	58 (8.7)	9 (5.5)	0.237	
Other (%)	88 (10.6)	68 (10.2)	20 (12.3)	0.537	
Isolated Brain injury (%)	266 (32.0)	238 (35.7)	28 (17.2)	<0.001	
White blood count - ×10^9^ cells/L (median [IQR])	12.5 [8.9, 16.7]	12.60 [9.20, 16.70]	12.0 [8.12, 17.17]	0.176	26
Haemoglobin - g/dl (median [IQR])	12.50 [10.60, 14.07]	12.60 [10.90, 14.10]	11.90 [9.75, 13.95]	0.014	24
Platelet count - ×10^9^ cells/L (median [IQR])	184 [133, 237]	188 [138, 240]	164 [112, 219]	0.002	25
Red blood cell distribution width - % (median [IQR])	13.7 [13.0, 14.5]	13.5 [13.0, 14.3]	14.20 [13.5, 15.3]	<0.001	25
Packed red blood cells - units (median [range])	0 [0–42]	0 [0–36]	0 [0–42]	<0.001	0
APTT ratio (median [IQR])	1.00 [0.90, 1.10]	1.00 [0.90, 1.10]	1.10 [0.90, 1.30]	<0.001	28
International normalised ratio (median [IQR])	1.10 [1.00, 1.20]	1.10 [1.00, 1.10]	1.10 [1.00, 1.20]	<0.001	31
Creatinine serum - µmol/L (median [IQR])	80 [65, 101]	78 [64, 95]	102 [74, 131]	<0.001	7
Urea serum - mmol/L (median [IQR])	4.8 [3.7, 6.3]	4.70 [3.6, 6.0]	5.7 [4.3, 7.6]	<0.001	7
Phosphate - mmol/L (median [IQR])	1.10 [0.87, 1.37]	1.07 [0.85, 1.31]	1.35 [1.00, 1.76]	<0.001	17
Calcium - mmol/L (median [IQR])	2.02 [1.90, 2.15]	2.03 [1.90, 2.15]	2.00 [1.87, 2.15]	0.305	16
Amylase - unit/L (median [IQR])	75 [45, 129]	72 [45, 121]	82.50 [46, 180]	0.008	30
Albumin - g/L (median [IQR])	37 [32, 42]	38 [33, 42]	35 [30, 40]	<0.001	11
C-reactive protein serum - mg/L (median [IQR])	8 [5, 39]	7 [5, 37]	14 [5, 60]	0.024	21
Alanine aminotransferase serum - unit/L (median [IQR])	36 [22, 78]	34 [21, 72]	51 [27, 120]	<0.001	12
Total bilirubin serum - µmol/L (median [IQR])	10 [7, 18]	10 [7, 17]	12 [8, 24]	0.001	12
Creatine kinase serum - unit/L (median [IQR])	770 [297, 1741]	708 [279, 1524]	1179 [424, 2790]	<0.001	16
ED lactate - mmol/L (median [IQR])	2.5 [1.7, 4.4]	2.5 [1.6, 3.8]	3.1 [2.0, 6.6]	<0.001	177
ED systolic blood pressure - mmHg (median [IQR])	124 [102, 145]	125 [104, 148]	120 [90, 140]	0.028	67
Radiological contrast in first 24 h (%)	613 (73.9)	494 (74.1)	119 (73.0)	0.86	
Hospital length of stay - days (median [IQR])	19 [8, 39]	18 [8, 38]	21 [8, 45]	0.317	
ICU length of stay - days (median [IQR])	5.2 [1.9, 11.4]	4.6 [1.8, 10.8]	7.2 [3.0, 14.5]	<0.001	
Hospital mortality (%)	156 (18.8)	103 (15.4)	53 (32.5)	<0.001	

Sites of injury were defined as an abbreviated injury scale score of >2 in up to three body sites. Continuous parameters are presented as median [Inter quartile range] and categorical parameters are presented as n (%). AKI acute kidney injury, SAPS-2 simplified acute physiology score two, APAC*HE II* acute physiology and chronic health evaluation II*, NISS* new injury severity score*, ISS* injury severity score*, APTT* activated partial thromboplastin time*, ED* emergency department*, ICU* intensive care unit.

**Figure 2:**
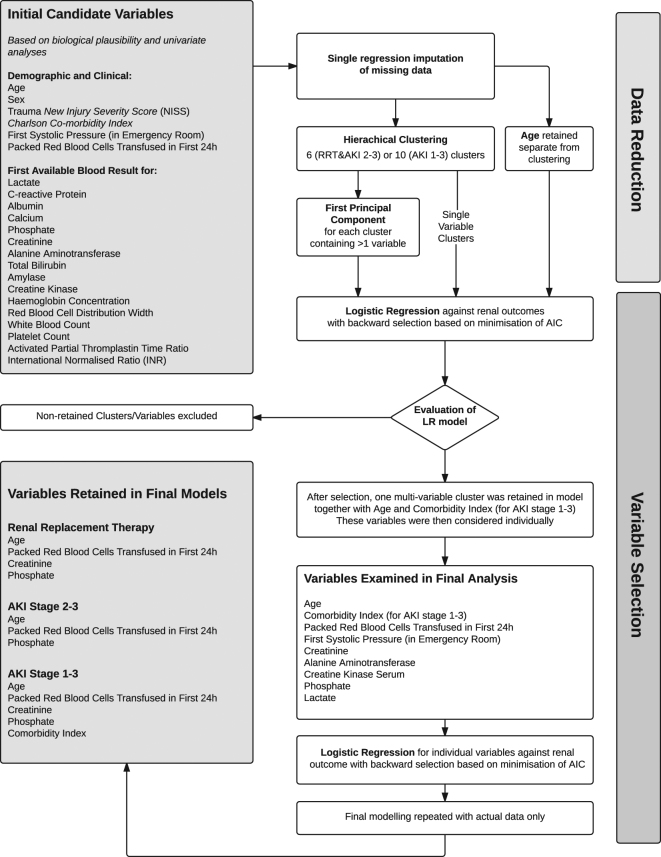
Process of variable selection and modelling for prediction of AKI outcomes.

**Figure 3:**
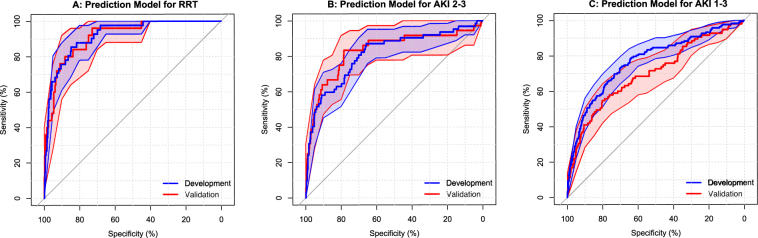
Receiver operating characteristic curve for prediction of renal replacement therapy (**A**), acute kidney injury stage 2–3 (**B**) and all acute kidney injury (**C**). Corresponding calibration plots available in supplement, Fig. S3a,b,c.

Variable selection and modelling is detailed in the supplementary appendix and summarised in Fig. 2. In brief, for prediction of RRT five clusters were identified; of these, only age and the first principal component of one cluster were significantly associated with RRT. A model was then developed by backward selection from the individual predictor variables within this cluster (ED systolic blood pressure, ED lactate, PRBC’s transfused in the first 24 h, and first ALT, CK, SCr, and phosphate) together with age. First SCr, first serum phosphate and number of PRBC’s were retained in the final model (Table 2, Fig. S3a). Modelling the development of moderate-severe (stage 2–3) AKI and any AKI (stage 1–3) was performed and the same predictors were included in the final models, except the omission of creatinine from the model for AKI stage 2–3 and the inclusion of Charlson comorbidity index in the model for any AKI. The model predicting need for RRT performed excellently with c-statistic of 0.92 (95% CI 0.88–0.96) – Fig. 3A ^18^. However, performance was progressively worse for models predicting moderate-severe (stage 2–3) AKI and for any AKI with c-statistics of 0.81 (0.75–0.88) and 0.77 (0.72–0.81), respectively (Table 2, Fig. 3B and C).

**Table 2:** Predictive models for AKI and RRT.

	Model for RRT*		Model for AKI 2–3*		Model for all AKI*	
	*Odds Ratio* *or c-Statistic* *(95% CI)*	*p-value*	*Odds Ratio* *or c-Statistic* *(95% CI)*	*p-value*	*Odds Ratio* *or c-Statistic* *(95% CI)*	*p-value*
Age (y)	1.031 (1.012–1.051)	0.0007	1.029 (1.014–1.044)	00001	1.020 (1.010–1.031)	0.0001
First Phosphate (mmol/L)	4.56 (2.48–8.40)	<0.0001	4.18 (2.54–6.89)	<0.0001	2.19 (1.46–3.29)	0.0002
First Creatinine (μmol/L)	1.009 (1.003–1.015)	0.027	*Not retained in model*		1.008 (1.003–1.0125)	0.0017
PRBC’s in first 24 h (Units)	1.121 (1.071–1.175)	0.0007	1.098 (1.054–1.143)	<0.0001	1.085 (1.046–1.125)	<0.0001
Charlson Index 1–2 (Reference 0)	*Not retained in model*		*Not retained in model*		2.36 (1.51–3.68)	0.0002
Charlson Index ≥3 (Reference 0)	*Not retained in model*		*Not retained in model*		2.92 (1.35–6.31)	0.0066
c-statistic *Development*	0.92 (0.88–0.96)	<0.0001	0.81 (0.75–0.88)	<0.0001	0.77 (0.72–0.81)	<0.0001
c-statistic *Validation*	0.91 (0.86–0.97)	<0.0001	0.83 (0.74–0.92)	<0.0001	0.70 (0.64–0.77)	<0.0001

*RRT* renal replacement therapy, *AKI* acute kidney injury, *PRBC* packed red blood cells. *Full specifications of model in supplementary material.

For validation, we identified 564 patients with incidence of AKI (17.4%), use of RRT (4.4%), hospital mortality (18.3%) and distribution of predictor variables comparable to our development dataset (Table S1). Performance of the models for RRT and AKI 2–3 prediction was similar in the validation cohort with c-statistics 0.91 (0.86–0.97) and 0.83 (0.74–0.92), respectively; however, the model for any AKI performed worse in the validation cohort (c-statistic 0.70, 0.64–0.77) – Table 2, Fig. 3A,B,C.

Despite apparently excellent discrimination of RRT, positive predictive value (PPV) at an optimal cut-off was 23.0% (13.7–42.7) in development and 20.7% (12.9–39.6) in validation although negative predictive value (NPV) was >99% (Table S2). However, a number of ‘false positive’ cases died, PPV for the alternative endpoint of RRT and/or death at the same cut-offs were 41.2% (34.8–48.1) and 50.5% (42.9–59.2) in development and validation, respectively (Table S2). Similar results were seen for prediction of AKI 2–3 and any AKI (Table S2).

CART analysis based on the variables identified in logistic regression demonstrated that large volume blood transfusion (≥14–16 units) was the most important determinant of risk for AKI and suggested simple cut-offs to identify groups at both high and low risk (Figs S13–S15). Overall CART models defined groups with higher PPV for AKI and the composite of death and/or, at the expense of somewhat lower sensitivity (Table S3).

## Discussion

### Main findings

From extensive demographic, biochemical and physiological data available close to ICU admission we identified, first serum phosphate, transfusion requirement in the first 24 h, age and first SCr as independent predictors of RRT, AKI stages 2–3, and any AKI within 7 days of major trauma requiring ICU admission. We demonstrated good to excellent discrimination of need for RRT in both development and validation cohorts and good discrimination of a wider group of patients with moderate-severe AKI (stage 2–3). Despite the high AUC values, PPVs for RRT or moderate-severe AKI were low at around 20%, this may reflect the relative infrequency of these endpoints so that even after significant enrichment incidence is low. In keeping with this, despite worse AUC the PPV for the any AKI outcome at the optimal cut-off was higher (Table S2). In addition, death may be an important and clinically relevant competing endpoint for development of AKI and need for RRT, and many “false positive” patients categorised as at risk of AKI stage 2–3 or RRT who did not achieve these endpoints in fact died with PPVs for a composite endpoint of death or AKI approaching 50% (Table S2). This suggests that patients identified by this model are a very high-risk population where the investigation of AKI-targeted therapy such as the early administration of RRT or adherence to “KDIGO bundles” is justified.

To our best knowledge this is the first prognostic model, specific to major trauma patients, to discriminate the need for RRT and development of AKI stage 2–3. Importantly, stage 2–3 AKI was associated with increased length of stay and independently predicted hospital mortality after adjustment for age and both trauma and critical illness severity. Improving early prediction of acute RRT and AKI stage 2–3 is likely to be necessary for effective evaluation of targeted interventions to reduce AKI-associated morbidity and mortality^8,19–21^.

### Comparison with existing literature

In the development cohort, we reported an incidence of AKI of 19.6% in a predominantly male (81.4%) population with a median age of 42 (IQR 24–50), limited comorbidity (Charlson comorbidity index of 0 in 76.5%) and who experienced major trauma (median injury severity score 25, IQR 17–33). The incidence of AKI in previous trauma-ICU studies ranges between 6 to 37%^1,2,12,22,23^, reflecting differing patient populations and ICU admission criteria. The incidence of RRT in our development and validation cohorts was 5.1% and 4.4% respectively, similar to the 4–7% incidence reported by other investigators^2,11,22–25^. In our study, stage 2–3 AKI was associated with increased mortality and increased length of hospital stay. In comparable studies of critically ill trauma patients, mortality ranged from 9.2 to 36%^1,2,22,23^. In a similar size cohort Biharoc^23^ demonstrated an independent association of maximal RIFLE-Risk AKI after trauma and mortality which was not evident in our study. This may have been due to the inclusion of less severe AKI defined by a 26.5 µmol/L SCr when using the KDIGO criteria rather than RIFLE as well as differences in definition of baseline SCr^20,23^. There was a similar exposure to radiological contrast in the first 24 hours between the AKI and non-AKI groups, Table 1.

The development model harnessed a broad range of variables available around ICU admission including many associated with AKI in trauma and general ICU cohorts^1,6,23,24,26–28^. In this study, Phosphate and SCr (for RRT and any AKI only) remained included in the final predictive logistic models for AKI. Phosphate is released by injured cells and is excreted by the kidney and might thus represent a marker of cellular injury after major trauma that precedes renal and multi-organ dysfunction. McMahon *et al*. incorporated phosphate as a significant variable into a risk prediction tool assessing likely need for RRT in rhabdomyolysis of mixed aetiology^29^, but to our knowledge this is the first description in a solely trauma population. Other prediction models for AKI have incorporated initial SCr in the settings of rhabdomyolysis, cardiac surgery and a general critical care cohorts^6,29,30^. In our study, SCr in univariate analysis was not strongly associated with AKI (Table 2), but was retained in models for RRT and any AKI in combination with other variables. Patients requiring a high number of PRBCs were at highest risk of developing severe AKI, consistent with the findings of several other major trauma studies^12,23,24^. While this may represent an association between transfusion and more severe injury, haemodynamic compromise or increased transfusion associated potassium load, the dominant impact of blood transfusion over other measures of severity suggests additional transfusion specific risk factors including transfusion related immunosuppression with greater risk of infection^31^ and/or exposure to products of haemolysis, plasma free haem and iron^32,33^. In contrast to other studies^34^, we did not associate serum creatine kinase levels with AKI, this may reflect the more multifactorial aetiology of AKI in our population. In addition, peak creatine kinase occurs 24–48 h after trauma, potentially reducing its prognostic significance when assessed immediately at hospital admission. Compared to other AKI risk prediction studies in other groups of critically ill patients, our models performed well. The c-statistics/AUCs of our models predicting RRT and moderate to severe AKI are comparable to the performance of the best novel AKI biomarkers^35^. In the analogous context of cardiac surgery, use of AKI biomarkers with this performance has enabled early intervention through the utilisation of a “KDIGO bundle” which included; optimisation of volume status and haemodynamics, avoidance of nephrotoxins and preventing hyperglycaemia to modify clinical outcomes^36^.

### Study Implications

While performance of logistic regression models for RRT and moderate to severe AKI was very good, accurate assessment of probability of AKI may not translate into accurate identification of individual clinical diagnoses. As described previously, despite apparently well-performing models, PPVs for RRT and AKI stages 2–3 were only around 20% (Table S2) but improved considerably, approaching 50% for the composite endpoint of death or AKI. This suggests our models do discriminate a population at very high risk of adverse outcome but the overall low frequency of RRT use limited its performance to the extent that, while apparently highly predictive, one would not be confident in directing therapy on the basis of these predictions beyond a trial setting. Similar studies on AKI prediction report comparable PPVs in the range of 27 to 45%^6,26,29^. Furthermore, application of a single cut-off to logistic regression models that provide a continuous probability estimate may not represent the optimum way to discretely categorise risk. CART models are well suited to the development of clinically interpretable models, but are vulnerable to over-fitting and may lack generalization^17^. In our complete dataset, CART models confirmed high volume transfusion as the most important predictor of AKI outcomes and better-categorised high-risk groups, suggesting that this approach could be useful in development of easily applicable predictive models from larger datasets (Table S3).

### Strengths and weaknesses

Our study has several strengths, we studied a large cohort of major trauma patients employing temporally separated development and validation datasets. We examined a wide range of variables close to ICU admission, but used data-reduction techniques to avoid over-fitting in subsequent predictive modelling. The final variables included in our model are also routinely available across other ICUs increasing the ease of future external validation.

There are some limitations to this work. As a single centre study, site and nature of injuries, pre-hospital care and demographics may not be generalisable to other settings. Similarly, there is considerable variability between institutions and clinicians regarding the initiation of RRT which further emphasises the need for external validation of this work. As discussed previously, the infrequency of RRT as an endpoint resulted in the low PPVs of the model and likely impacted the model calibration, Fig. S7. AKI diagnoses were based on SCr and/or need for RRT, without urine output information which may have resulted in missed AKI diagnoses. In the absence of true baseline SCr in almost all trauma patients, we used the first recorded value in hospital as baseline for AKI assessment – possibly leading to misclassification of AKI. However, estimates of baseline, as used by other investigators, is likely to under-estimate true baseline GFR in a younger trauma population^37^, while a raised SCr that only falls after admission is likely to be a transient form of AKI, which is not directly comparable to that related to a rising SCr in hospital. We were less successful in discriminating AKI stage 1–3 and this may reflect the multifactorial nature of AKI-risk in the trauma population including some risk-factors which only accrue after ICU admission. Major blood transfusion was very important in our models, this information was only available as total over the first 24 h after trauma not strictly at ICU admission. However, in our experience the vast-majority of these products are provided in the ED and the operating theatre prior to ICU. The Charlson comorbidity index was calculated from encounters at the Royal London Hospital only and therefore may have underestimated the presence of comorbidities. Despite this, the low level of comorbidity recorded is in keeping with the relatively young, trauma population. Finally, other potential AKI risk-factors could not be quantified, either because exposure was near universal (such as radiological contrast related to trauma-series CT scans), or were not measured in our population (such as pre-ICU admission fluid balance, serum myoglobin, or novel AKI biomarkers). Our study thus represents only a pragmatic assessment of available clinical data in a real-world context and a benchmark to assess additional diagnostic techniques including novel biomarkers. Finally, other methods such as CART modelling may improve our discrimination of this high-risk group and provide easy to use decision rules, however they are prone to over-fitting and require larger datasets to explore fully.

## Conclusions

The development of moderate-severe AKI and need for RRT can be predicted from demographic, clinical and biochemical data acquired routinely close to ICU admission. If generalized across other centres, such models could provide a tool to target existing and novel approaches for treatment or prevention of AKI immediately after trauma ICU admission. This will increase chances of clinical benefit, as well as the power and feasibility of clinical trials and provides greater confidence and equipoise to investigate novel AKI targeted therapies.

### Data Sharing

Access to fully anonymised datasets is available on request to the corresponding author, subject to submission of a proposal and data analysis plan.

## Electronic supplementary material

Supplementary Material
