# Supplementary material for: Acute Kidney Injury in Trauma Patients Admitted to Critical Care: Development and Validation of a Diagnostic Prediction Model
Source: Sci Rep. 2018 Feb 26;8:3665. doi: 10.1038/s41598-018-21929-2 (PMC5827665; doi:10.1038/s41598-018-21929-2)
Supplement: Supplementary file 1 — Supplementary Material [file 41598_2018_21929_MOESM1_ESM.pdf]

Acute Kidney Injury in Trauma Patients Admitted to Critical Care: Development and Validation of a Diagnostic Prediction Model

Supplementary Material

Ryan W. Haines*[1,2], Shih-Pin Lin*[3,4], Russell Hewson[1,2], Christopher J. Kirwan[1,2,6], Hew Torrance[1,2,5], Michael O'Dwyer[1,2], Anita West[5], Karim Brohi[5], Rupert Pearse[1,2], Parjam Zolfaghari[1,2], John R. Prowle[1,2,6]

[1]Adult Critical Care Unit, The Royal London Hospital, Barts Health NHS Trust, Whitechapel Road, London, E1 1BB, UK

[2]William Harvey Research Institute, Queen Mary University of London, London, UK

[3]Department of Anesthesiology, Taipei Veterans General Hospital and School of Medicine, National Yang-Ming University, Taipei, Taiwan

[4]Division of Biostatistics, Graduate Institute of Epidemiology and Preventive Medicine, College of Public Health, National Taiwan University

[5]Centre for Trauma Sciences, Blizard Institute, Queen Mary University of London, UK

[6]Department of Renal Medicine and Transplantation, The Royal London Hospital, Barts Health NHS Trust, Whitechapel Road, London, E1 1BB, UK

Table S1. Baseline characteristics of development and validation cohorts comparing acute kidney injury with no acute kidney injury.

	Development				Validation			
	All patients	No AKI	AKI 1-3	p-value AKI vs no AKI	All patients	No AKI	AKI 1-3	p-value AKI vs no AKI
Number (%)	830 (100)	667 (80.4)	163 (19.6)	-	564 (100)	466 (82.6)	98 (17.4)	-
Age - years (IQR)	42 (24-50)	40 (27-54)	50 (32-66)	0.4	41 (27-58)	40 (25-56)	52 (33-71)	<0.001
Male sex (%)	676 (81.4)	539 (80.8)	137 (84.0)	<0.001	437 (77.5)	361 (77.5)	76 (77.6)	1.0
NISS (IQR)	34 (24-50)	34 (22-50)	34 (25-50)	0.66	34 (22-50)	34 (21-50)	43 (29-57)	<0.001
Hospital LOS - *days* (IQR)	19 (8-39)	18 (8-38)	21 (8-45)	0.32	15 (7-32)	15 (7-31)	19 (7-37)	0.44
Hospital mortality (%)	156 (18.8)	103 (15.4)	53 (32.5)	<0.001	103 (18.3)	63 (13.5)	40 (40.8)	<0.001
RRT (%)	42 (5.1)	-	42 (25.8)	-	25 (4.4)	-	25 (25.5)	-
AKI 2-3 (%)	65 (7.8)	-	65 (40.0)	-	31 (5.5)	-	31 (31.6)	-
Phosphate - *mmol/L*	1.10 (0.87-1.37)	1.07 (0.85-1.31)	1.35 (1.00-1.76)	<0.001	1.13 (0.91-1.37)	1.12 (0.91-1.37)	1.27 (0.99-1.55)	0.002
Creatinine - *µmol/L*	80 (65-101)	78 (64-95)	102 (74-131)	<0.001	82 (65-100)	81 (65-98)	87 (66-114)	0.098
PRBCs - *units*	0 (0-42)	0 (0-36)	0 (0-42)	<0.001	0 (0-50)	0 (0-19)	0 (0-50)	<0.001
CCI 0	635 (76.5)	540 (81.0)	95 (58.3)		389 (71.6)	333 (74.3)	56 (58.9)	
CCI 1-2	159 (19.2)	106 (15.9)	53 (32.5)	<0.001	91 (16.8)	71 (15.8)	20 (21.1)	0.004
CCI ≥3	36 (4.3)	21 (3.1)	15 (9.2)		63 (11.6)	44 (9.8)	19 (20.0)	

Median (interquartile range-IQR) or number (%). *CCI Charlson comorbidity index, NISS* new injury severity score, *LOS* length of stay, *RRT* renal replacement therapy.

Table S2. Development and validation model diagnostic parameters.

	RRT		AKI 2-3		All AKI	
	Develop	Valid	Develop	Valid	Develop	Valid
Prevalence of AKI outcome	5.10% (3.6-6.5)	4.40% (2.8-6.2)	7.60% (5.8-9.4)	6.30% (4.4-8.3)	19.60% (17.0-22.4)	17.40% (14.1-20.6)
Model c-stat / AUC	0.92 (0.88-0.96)	0.91 (0.86-0.97)	0.81 (0.75-0.88)	0.83 (0.74-0.92)	0.77 (0.73-0.81)	0.7 (0.64-0.77)
Optimal cut-off	4.90% (2.3-10.2)	4.30% (2.6-9.6)	5.90% (5.5-12.9)	7.30% (5.0-12.2)	17.60% (14.7-28.4)	25.20% (16.4-36.1)
Sens	87.80% (75.6-100)	92.00% (76.0-100)	83.90% (58.1-93.5)	83.30% (66.7-94.4)	72.80% (55.5-84.0)	56.80% (40.0-72.6)
Spec	84.30% (67.4-94.5)	84.10% (70.4-94.2)	68.80% (63.2-93.2)	79.10% (67.6-92.3)	72.00% (61.8-88.2%)	80.20% (63.4-92.1)
PPV	23.0% (13.7-42.7)	20.7% (12.9-39.6)	18.8% (16.0-43.0)	21.8% (16.3-39.7)	39.6% (33.9-54.6)	38.4% (28.8-54.2)
NPV	99.3% (98.6-100)	99.5% (98.8-100)	98.2% (96.3-99.2)	98.5% (97.4-99.5)	91.5% (88.6-94.2)	89.7% (87.3-92.2)
Accuracy	85% (69-94)	84% (71-94)	70% (65-91)	79% (69-91)	72% (65-82)	76% (65-84)
PPV for AKI or Death	41.2% (34.8-48.1)	50.5% (42.9-59.2)	34.7% (30.6-38.9)	46.3% (40.0-53.2)	49.2% (45.2-53.2)	53.6% (46.9-60.5)

RRT renal replacement therapy, *AKI* acute kidney injury, *AUC area under the curve, PPV positive predictive value, NPV negative predictive value.*

Table S3: Prediction of AKI in the whole patient cohort (development and verification) based on Classification and Regression Tree (CART) modelling utilizing the predictor variables identified in regression modelling. *RRT* renal replacement therapy, *AKI* acute kidney injury, *AUC area under the curve, PPV positive predictive value, NPV negative predictive value.*

	**RRT**	**AKI 2-3**	**Any AKI**
Prevalence of AKI outcome	4.80% (3.7-6.0)	7.11% (5.8-8.5)	18.7% (16.7-20)
Cut-off used (terminal node probability)	≥0.35	≥0.40	≥0.56
Regression tree AUC	0.83 (0.78-0.89)	0.72 (0.67-0.77)	0.74 (0.71-0.77)
Sens	70.1% (59.7-80.6)	45.5% (36.4-55.6)	39.9% (34.1-45.6)
Spec	95.3% (94.2-96.5)	97.1% (96.1-97.9)	94.5% (93.2-95.8%)
PPV	43.1% (36.3-51.0)	54.3% (45.1-63.6)	62.7% (56.2-69.4)
NPV	98.4% (97.9-99.0)	95.7% (95.0-96.4)	87.2% (86.1-88.3)
Accuracy	94.1% (93.0-95.3)	95.9% (95.2-96.6)	84.29% (82.7-85.6)
PPV for AKI or Death	63.3% (54.6-71.7)	69.8% (60.0-79.2)	75.7% (74.1-77.3)

Figure S1. Development cohort consort diagram. *ED* emergency department, *ICU* intensive care unit.

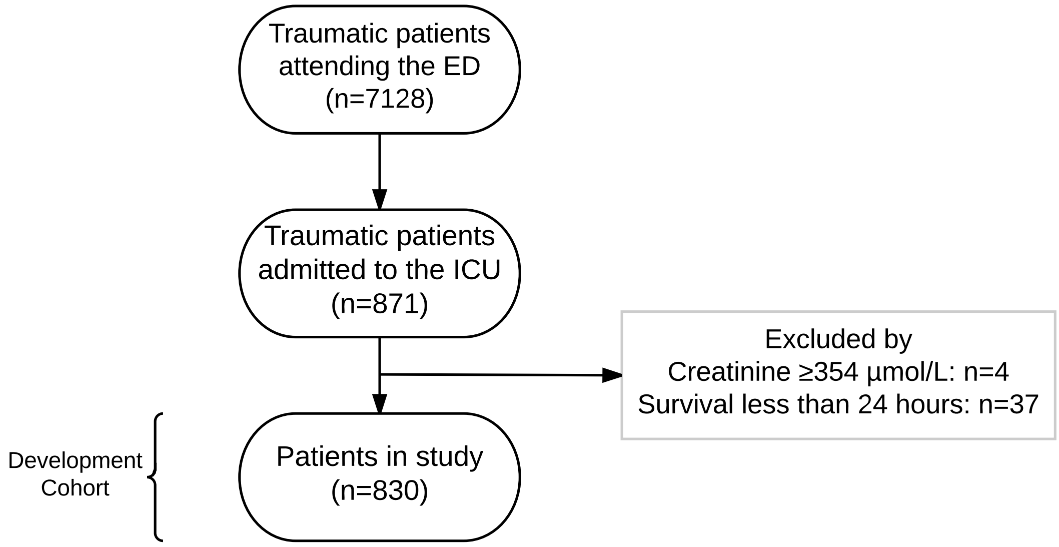

Figure S2. Logistic Regression for hospital mortality. For continuous covariates Odds Ratios represent comparison of 25th centile vs 75th centile. Different shaded areas of bars indicate different confidence levels (0.9, 0.95, 0.99). *SAPS II score calculated with subtraction of component related to highest serum Urea. AKI - Acute Kidney Injury, SAPS – Simplified Acute Physiology Score, NISS – New Injury Severity Score.

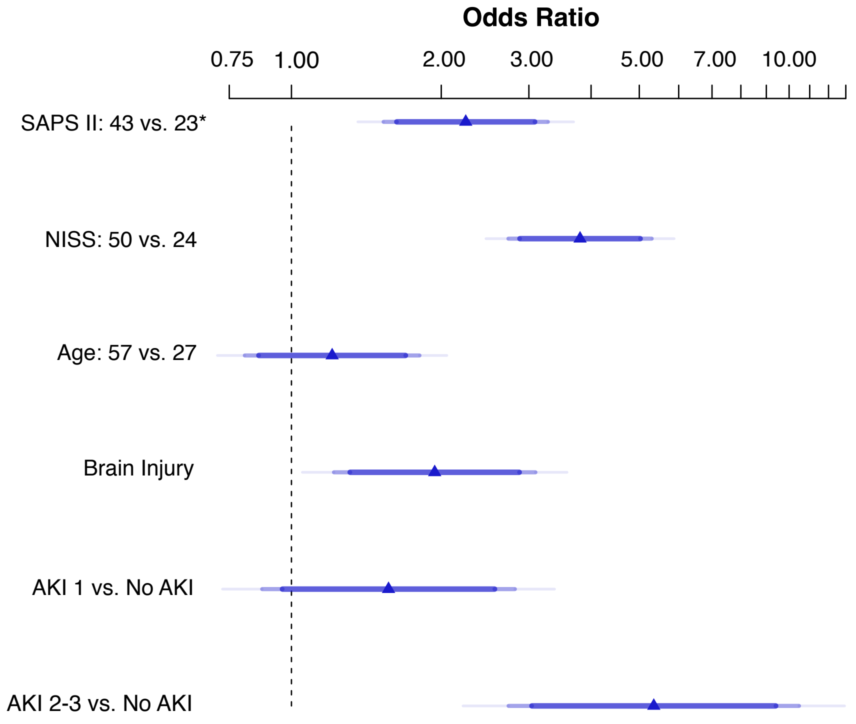

Figure S3. Odds ratio for individual model parameters for prediction of renal replacement therapy (a), acute kidney stage 2-3 (b) and all acute kidney injury (c). For continuous covariates Odds Ratios represent comparison of 25th centile vs 75th centile. Different shaded areas of bars indicate different confidence levels (0.9, 0.95, 0.99). *PO4* phosphate, *PRBC* packed red blood cells.

a: RRT

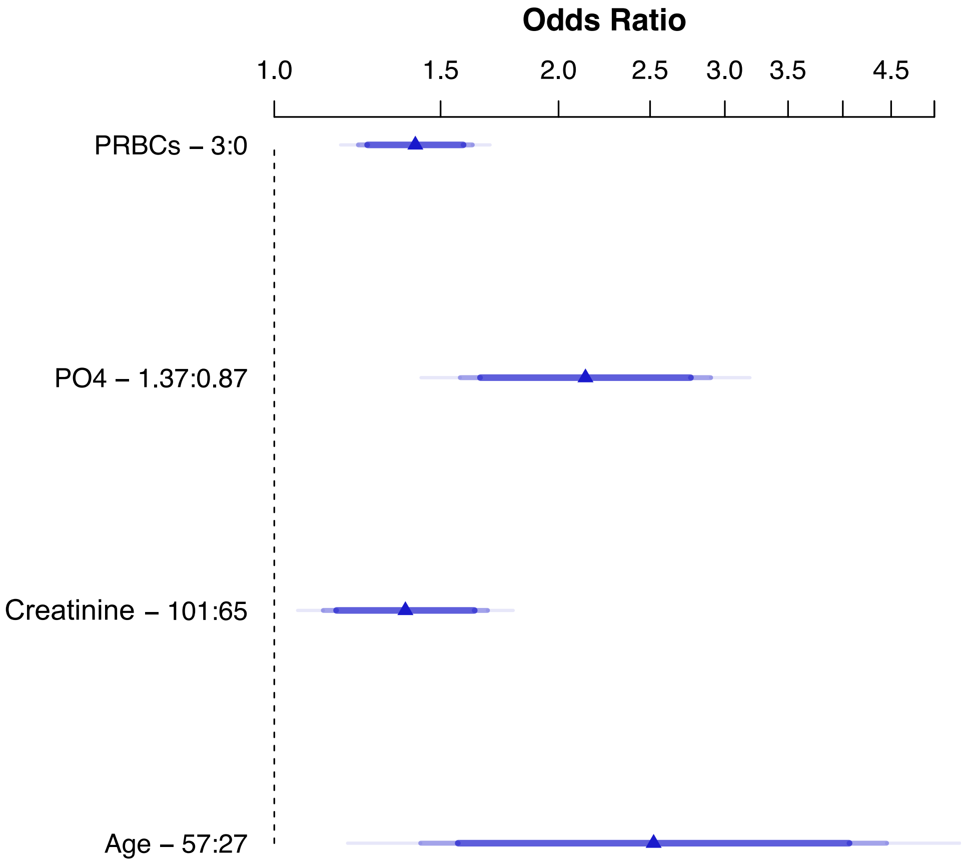

Odds Ratio
1.0
1.5
2.0
2.5
3.0
3.5
4.5
PRBCs – 3:0
PO4 – 1.37:0.87
Creatinine – 101:65
Age – 57:27

b: AKI 2-3

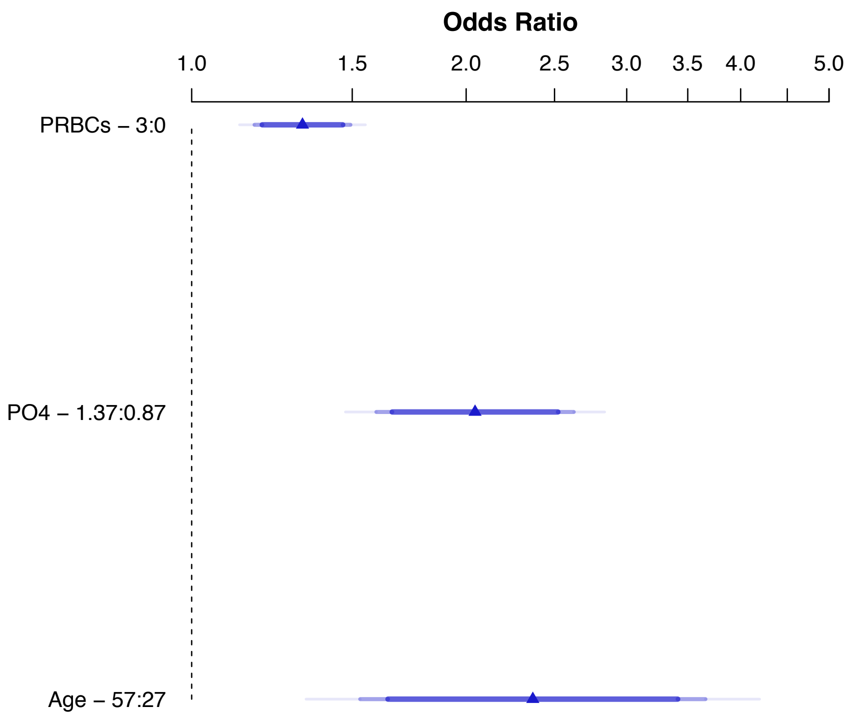

c: Any AKI

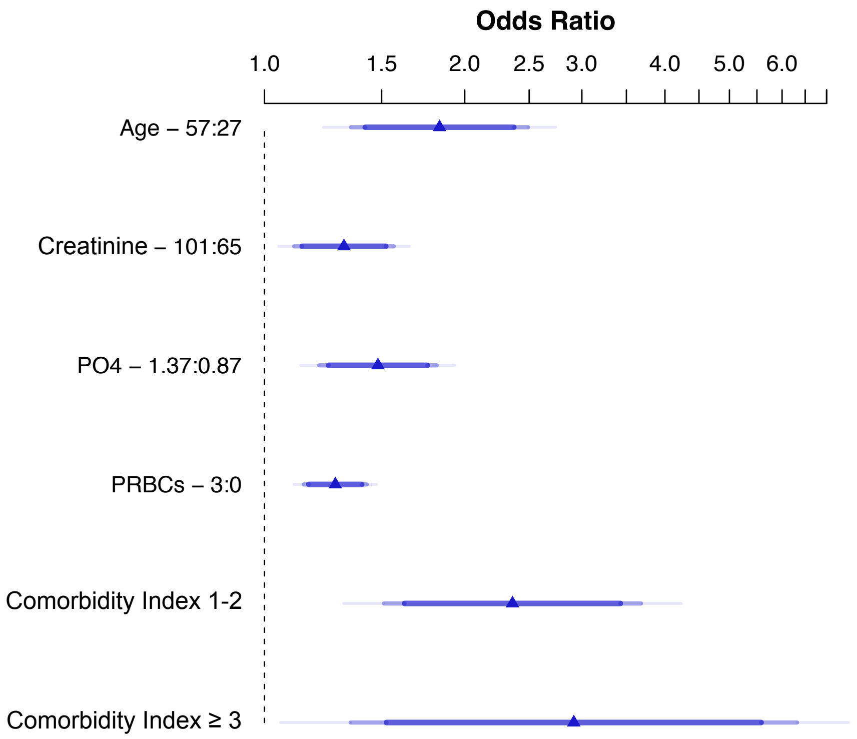

Statistical Analysis – Detailed description

The following R-packages were employed in data preparation analysis and presentation:

- *rms* Frank E Harrell Jr (2017). rms: Regression Modeling Strategies. R package version
- 5.1-0. https://CRAN.R-project.org/package=rms
- *ggplot2* H. Wickham. ggplot2: Elegant Graphics for Data Analysis. Springer-Verlag New York, 2009
- *readr* Hadley Wickham, Jim Hester and Romain Francois (2016). readr: Read Tabular Data. R package version 1.0.0. https://CRAN.R-project.org/package=readr
- *dplyr,* Hadley Wickham and Romain Francois (2016). dplyr: A Grammar of Data Manipulation. R package version 0.5.0. https://CRAN.R-project.org/package=dplyr
- *car,* John Fox and Sanford Weisberg (2011). An R Companion to Applied Regression, Second Edition. Thousand Oaks CA: Sage. URL:http://socserv.socsci.mcmaster.ca/jfox/Books/Companion
- *pROC* Xavier Robin, Natacha Turck, Alexandre Hainard, Natalia Tiberti, Frédérique Lisacek, Jean-Charles Sanchez and Markus Müller (2011). pROC: an open-source package for R and S+ to analyze and compare ROC curves. BMC Bioinformatics, 12, p. 77. DOI: 10.1186/1471-2105-12-77
- *rpart T*erry Therneau, Beth Atkinson and Brian Ripley (2015) rpart: Recursive Partitioning and Regression Trees. R package version 4.1-10. https://CRAN.R-project.org/package=rpart
- *icd.* Jack O. Wasey (2016). icd: Tools for Working with ICD-9 and ICD-10 Codes, and Finding Comorbidities. R package version 2.1. https://CRAN.R-project.org/package=icd

Data reduction

To handle the large number of candidate predictor variables without over-fitting, we undertook unsupervised data-reduction prior to model building [15]. Firstly, simultaneous transformation (variables normalized to have mean 0 and standard deviation 1) with single imputation was performed– Fig S4. This was followed by hierarchical cluster analysis of the transformed dataset using Hoeffding's D statistic as a similarity measure. As age was deemed important to include in the final model selection process, we excluded age from the cluster analysis. For prediction of RRT (42 events) and AKI 2-3 (63 events) 6 clusters were used (Fig S5), for prediction of any AKI (163 events) 10 clusters were selected (Fig S6).

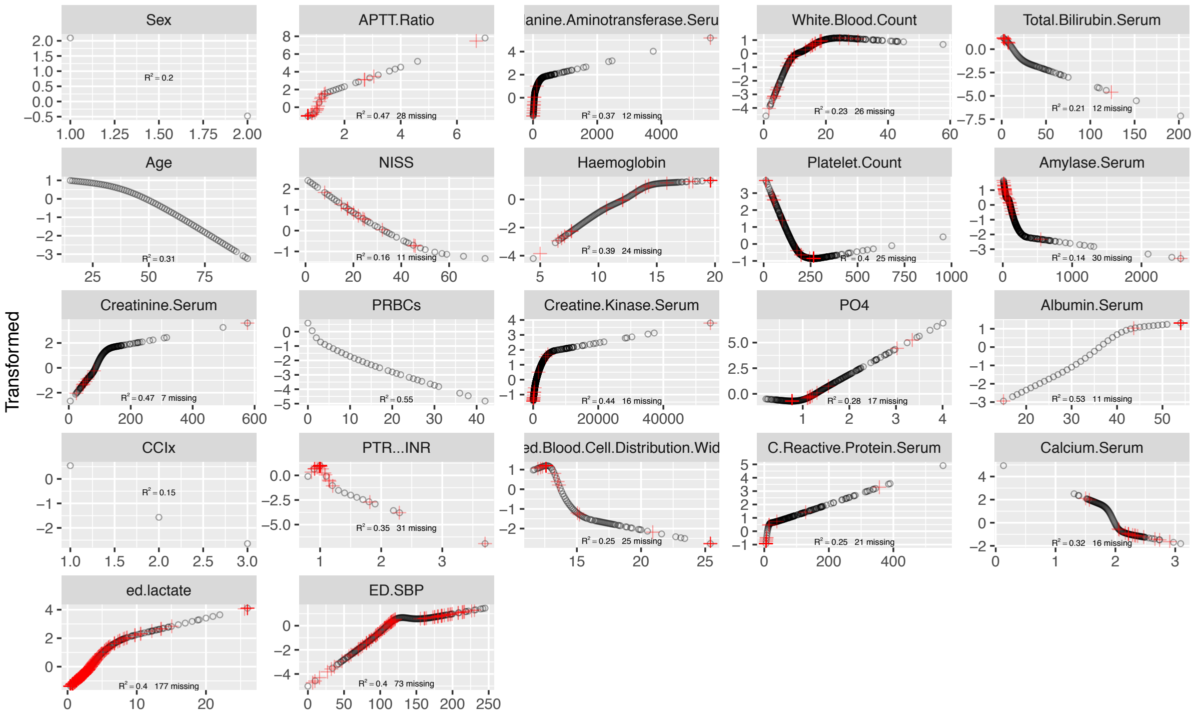

Figure S4: Simultaneous transformation and single imputation of potential predictor variables prior to cluster analysis using the R package *rms* function *transcan.*

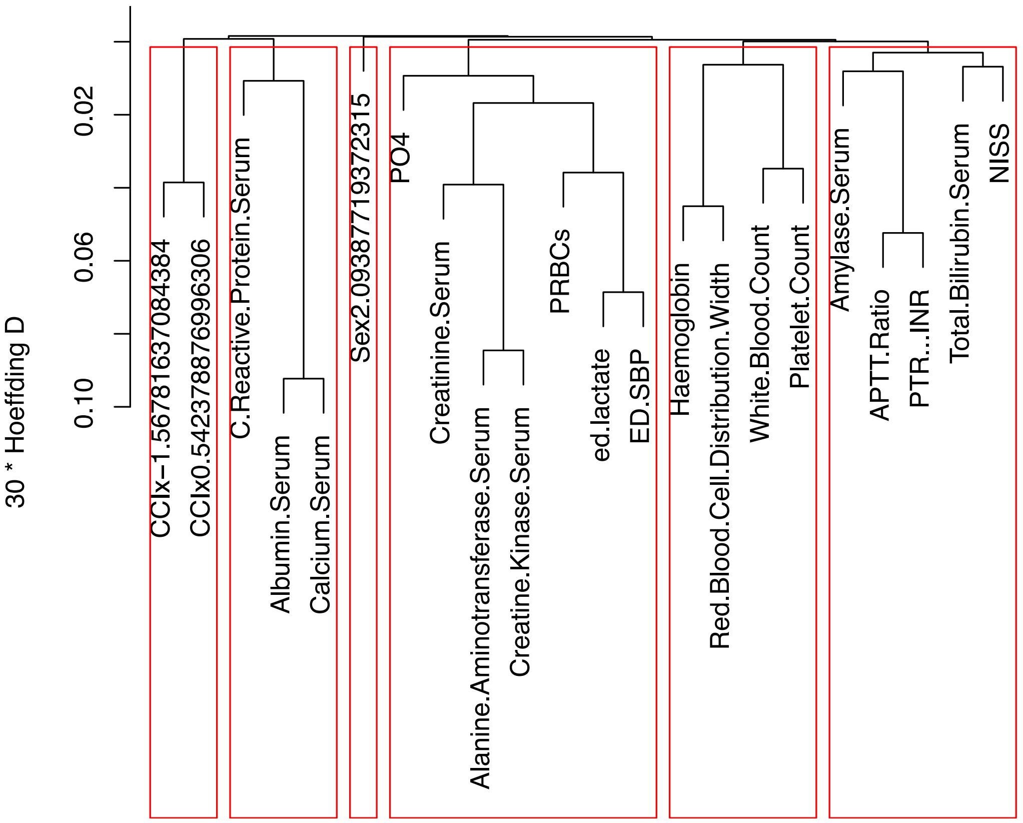

Figure S6: Hierarchical cluster analysis – 6 clusters

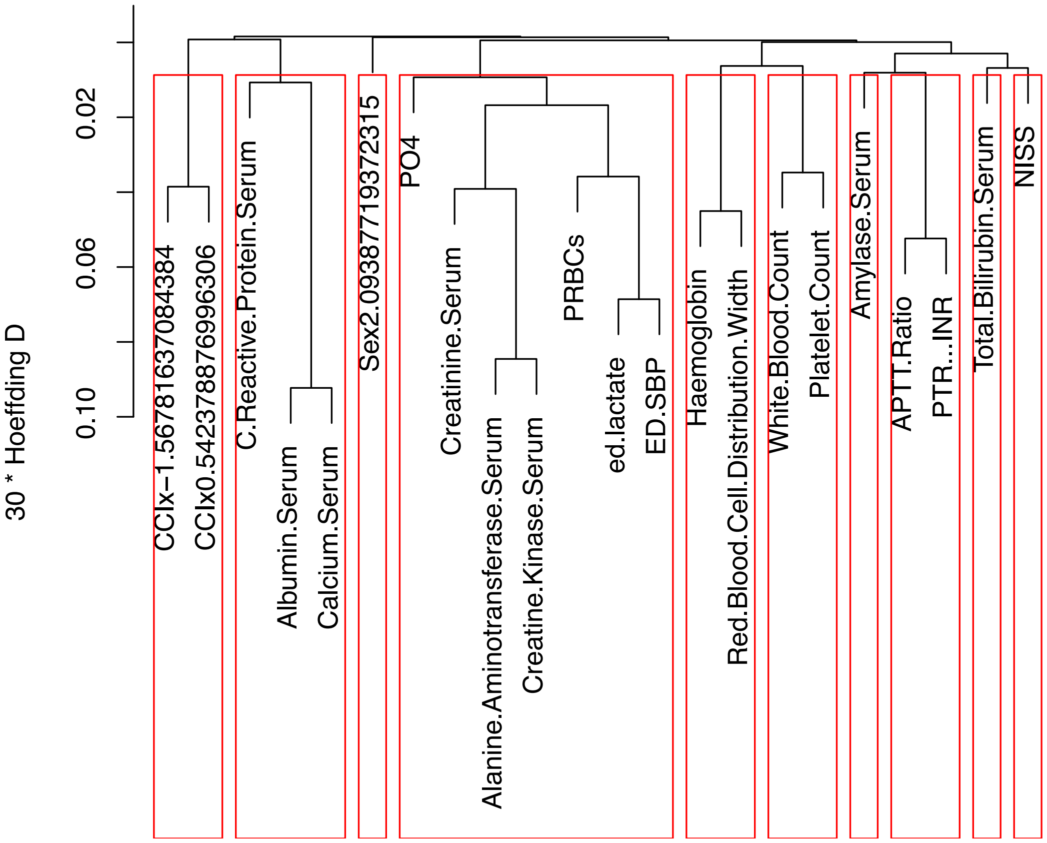

Figure S7: Hierarchical cluster analysis – 10 clusters

Model selection

For clusters containing >1 variable the first principal component of the transformed variables within the cluster was then extracted and analysed together with age and single variable clusters in logistic regression for prediction of AKI outcomes. This was followed by backward variable selection based on minimisation of the AIC (Tables S4 and S5). After backward selection only one cluster containing the variables: *Creatinine Serum, Alanine Aminotransferase Serum, Creatine Kinase Serum, Phosphate, Lactate, PRBCs transfused, Emergency Department Systolic Blood Pressure* was retained in the models, together with *Age* and, for all AKI prediction, *Comorbidity Index*.

Table S4: Variable selection based on principal components of hierarchical clusters: RRT and AKI 2-3.

Cluster or variable	Proportion of variance accounted for by PC1	RRT		AKI 2-3	
		p-value in initial logistic regression	Retained in model after selection	p-value in initial logistic regression	Retained in model after selection
Albumin.Serum, C.Reactive.Protein.Serum, Calcium.Serum	0.57	0.481	No	0.081	No
Creatinine.Serum, Alanine.Aminotransferase.Serum, Creatine.Kinase.Serum, PO4, ed.lactate, PRBCs, ED.SBP	0.48	<0.0001	Yes	<0.0001	Yes
White.Blood.Count, Platelet.Count, Haemoglobin, Red.Blood.Cell.Distribution.Width	0.51	0.713	No	0.589	No
Amylase.Serum, APTT.Ratio, PTR...INR, Total.Bilirubin.Serum, NISS	0.38	0.810	No	0.175	No
Age		0.001	Yes	0.0006	Yes
Sex (Male)		0.529	No	0.945	No
Charlson Index 1-2<br>Charlson Index ≥3		0.732<br>0.541	No	0.159<br>0.108	No

Table S5: Variable selection based on principal components of hierarchical clusters: Any AKI.

Cluster or variable	Proportion of variance accounted for by PC1	p-value in logistic regression for Any AKI	Retained in model after selection
Albumin.Serum, C.Reactive.Protein.Serum, Calcium.Serum	0.57	0.982	No
Haemoglobin, Red.Blood.Cell.Distribution.Width	0.71	0.333	No
Creatinine.Serum, Alanine.Aminotransferase.Serum, Creatine.Kinase.Serum, PO4,ed.lactate,PRBCs,ED.SBP	0.48	<0.0001	Yes
White.Blood.Count,Platelet.Count	0.70	0.498	Yes
APTT.Ratio, PTR...INR	0.77	0.227	No
Amylase.Serum		0.519	No
Total.Bilirubin.Serum		0.044	No
NISS		0.892	No
Age		<0.0001	Yes
Sex		0.128	No
Charlson Index 1-2<br>Charlson Index ≥3		0.0002<br>0.0086	Yes

Final models

As a single significant cluster was very strongly associated with the outcome variable we then considered all variables within this cluster together with Age (and comorbidity for modelling any AKI) in a further process of backward variable elimination to develop final logistic models containing individual variables. These models were then refitted to the variables in the original dataset without imputation (17 patients with missing data excluded including one case of AKI) to derive the final predictive models (Boxes S1-S6). Confidence interval (CI) of the coordinates of ROC curves (Table S2) were computed from 2000 stratified bootstrap replicates allowing CI estimates for thresholds, sensitivity, specificity, positive and negative predictive values using the R package *pROC*.

Box S1: Second stage variable selection: RRT

```
                               Coef    S.E.   Wald Z Pr(>|Z|)
 Intercept                     -9.8216 1.2104 -8.11  <0.0001
 Age                            0.0320 0.0100  3.20  0.0014
 Creatinine.Serum               0.0064 0.0028  2.28  0.0226
 Alanine.Aminotransferase.Serum 0.0002 0.0004  0.39  0.6933
 Creatine.Kinase.Serum          0.0000 0.0000 -0.03  0.9742
 PO4                            1.5631 0.3464  4.51  <0.0001
 PRBCs                          0.1463 0.0296  4.94  <0.0001
 ED.SBP                         0.0131 0.0054  2.45  0.0141
 ed.lactate                     0.0276 0.0496  0.56  0.5775


 Deleted                        Chi-Sq d.f. P      Residual d.f. P      AIC
 Creatine.Kinase.Serum          0.00   1    0.9742 0.00     1    0.9742 -2.00
 Alanine.Aminotransferase.Serum 0.15   1    0.6942 0.16     2    0.9252 -3.84
 ed.lactate                     0.56   1    0.4535 0.72     3    0.8691 -5.28
 ED.SBP                         5.71   1    0.0169 6.43     4    0.1695 -1.57

Approximate Estimates after Deleting Factors

                      Coef     S.E. Wald Z         P
Intercept        -7.622398 0.829442 -9.190 0.000e+00
Age               0.032064 0.009755  3.287 1.013e-03
Creatinine.Serum  0.005766 0.002705  2.131 3.305e-02
PO4               1.459528 0.310864  4.695 2.665e-06
PRBCs             0.113325 0.023930  4.736 2.183e-06
```

Box S2: Final Model for RRT

```
                       Model Likelihood     Discrimination    Rank Discrim.
                          Ratio Test           Indexes           Indexes
 Obs            813    LR chi2      109.10    R2         0.381    C         0.920
```

```
 0            772    d.f.             4    g        1.413    Dxy     0.841
 1             41    Pr(> chi2) <0.0001    gr       4.107    gamma   0.844
 max |deriv| 8e-10                         gp       0.071    tau-a   0.081
                                           Brier    0.036

                 Coef    S.E.   Wald Z Pr(>|Z|)
 Intercept      -8.0762 0.8371 -9.65  <0.0001
 PRBCs           0.1147 0.0236  4.86  <0.0001
 PO4             1.5175 0.3113  4.87  <0.0001
 Creatinine      0.0089 0.0028  3.13   0.0017
 Age             0.0308 0.0097  3.19   0.0014
```

Box S3: Second stage variable selection: AKI 2-3

```
                               Coef    S.E.   Wald Z Pr(>|Z|)
 Intercept                    -7.1976 0.8667 -8.30  <0.0001
 Age                           0.0297 0.0077  3.88  0.0001
 Creatinine.Serum              0.0051 0.0025  2.02  0.0439
 Alanine.Aminotransferase.Serum  0.0002 0.0003  0.63  0.5305
 Creatine.Kinase.Serum         0.0000 0.0000 -0.08  0.9392
 PO4                           1.1442 0.2817  4.06  <0.0001
 PRBCs                         0.0962 0.0246  3.92  <0.0001
 ED.SBP                        0.0055 0.0042  1.31  0.1893
 ed.lactate                    0.0343 0.0407  0.84  0.3988


 Deleted                        Chi-Sq d.f. P      Residual d.f. P      AIC
 Creatine.Kinase.Serum          0.01   1    0.9392 0.01     1    0.9392 -1.99
 Alanine.Aminotransferase.Serum 0.39   1    0.5335 0.39     2    0.8214 -3.61
 ed.lactate                     1.28   1    0.2588 1.67     3    0.6439 -4.33
 ED.SBP                         1.34   1    0.2470 3.01     4    0.5563 -4.99
 Creatinine.Serum               4.44   1    0.0352 7.44     5    0.1896 -2.56

Approximate Estimates after Deleting Factors

             Coef    S.E.  Wald Z         P
Intercept -6.03545 0.58599 -10.300 0.000e+00
Age        0.02913 0.00745   3.911 9.204e-05
PO4        1.35325 0.24926   5.429 5.664e-08
PRBCs      0.09202 0.02043   4.503 6.692e-06
```

Box S4: Final Model for AKI 2-3

```
                       Model Likelihood    Discrimination    Rank Discrim.
                          Ratio Test          Indexes           Indexes
 Obs            813    LR chi2      87.14    R2       0.244    C       0.813
  FALSE         751    d.f.             3    g        1.117    Dxy     0.626
  TRUE           62    Pr(> chi2) <0.0001    gr       3.056    gamma   0.630
 max |deriv| 5e-12                           gp       0.082    tau-a   0.088
                                             Brier    0.058

           Coef    S.E.   Wald Z Pr(>|Z|)
 Intercept -6.1423 0.5871 -10.46 <0.0001
 PRBCs      0.0933 0.0206   4.53 <0.0001
 PO4        1.4312 0.2542   5.63 <0.0001
 Age        0.0287 0.0074   3.87  0.0001
```

Box S5: Second stage variable selection: Any AKI

	Coef	S.E.	Wald Z	Pr(>|Z|)
Intercept	-4.5884	0.5611	-8.18	<0.0001
CCI 1-2	0.8418	0.2272	3.71	0.0002
CCI ≥3	1.1041	0.3882	2.84	0.0045
Age	0.0229	0.0054	4.23	<0.0001
Creatinine.Serum	0.0048	0.0022	2.15	0.0316
Alanine.Aminotransferase.Serum	0.0000	0.0003	-0.10	0.9181
Creatine.Kinase.Serum	0.0000	0.0000	-0.01	0.9924
PO4	0.6636	0.2163	3.07	0.0022
PRBCs	0.0767	0.0209	3.67	0.0002
ED.SBP	0.0010	0.0029	0.35	0.7276
ed.lactate	0.0574	0.0306	1.87	0.0609

Deleted	Chi-Sq	d.f.	P	Residual	d.f.	P	AIC
Creatine.Kinase.Serum	0.00	1	0.9924	0.00	1	0.9924	-2.00
Alanine.Aminotransferase.Serum	0.01	1	0.9153	0.01	2	0.9943	-3.99
ED.SBP	0.12	1	0.7292	0.13	3	0.9878	-5.87
ed.lactate	3.52	1	0.0607	3.65	4	0.4555	-4.35

Approximate Estimates after Deleting Factors

	Coef	S.E.	Wald Z	P
Intercept	-4.365111	0.394655	-11.061	0.000e+00
CCI 1-2	0.856390	0.225182	3.803	1.429e-04
CCI ≥3	1.110519	0.387886	2.863	4.196e-03
Age	0.020977	0.005207	4.029	5.602e-05
Creatinine.Serum	0.005454	0.002159	2.526	1.153e-02
PO4	0.766966	0.206455	3.715	2.033e-04
PRBCs	0.085067	0.018562	4.583	4.586e-06

Box S6: Final Model for Any AKI

		**Model Likelihood Ratio Test**		**Discrimination Indexes**		**Rank Discrim. Indexes**	
Obs	813	LR chi2	131.71	R2	0.237	C	0.768
FALSE	651	d.f.	6	g	1.099	Dxy	0.537
TRUE	162	Pr(> chi2)	<0.0001	gr	3.000	gamma	0.539
max |deriv|	4e-12			gp	0.166	tau-a	0.172
				Brier	0.130		

	**Coef**	**S.E.**	**Wald Z**	**Pr(>|Z|)**
Intercept	-4.5288	0.4056	-11.17	<0.0001
CCI 1-2	0.8586	0.2270	3.78	0.0002
CCI ≥3	1.0708	0.3942	2.72	0.0066
Age	0.0202	0.0052	3.88	0.0001
Creatinine	0.0076	0.0024	3.13	0.0017
PO4	0.7855	0.2074	3.79	0.0002
PRBCs	0.0817	0.0185	4.41	<0.0001

Model calibration and goodness of fit

Model calibration was assessed by calibration plots and tested with the Cessie and van Houwelingen global goodness of fit test on the development data (Fig S8-S10). Further calibration plots were prepared using the validation datasets (Fig S11-S13). Despite excellent c-statistic (0.92) there was evidence of significant deviation between actual and predicted probabilities for the model predicting RRT (p=0.003), however fit was reasonable over most of the range of observed probabilities with a low mean absolute error (Fig S8) and calibration was better on validation data (Fig S11) with low values for *Brier* and *Unreliability* statistics.

There was no evidence of significant departure from global goodness of fit for models predicting AKI 2-3 and any AKI. While the model for any AKI was least discriminating in terms of AUC/C-statistic it appeared best calibrated in terms of the relationship between actual and observed probabilities in both the development and validation datasets.

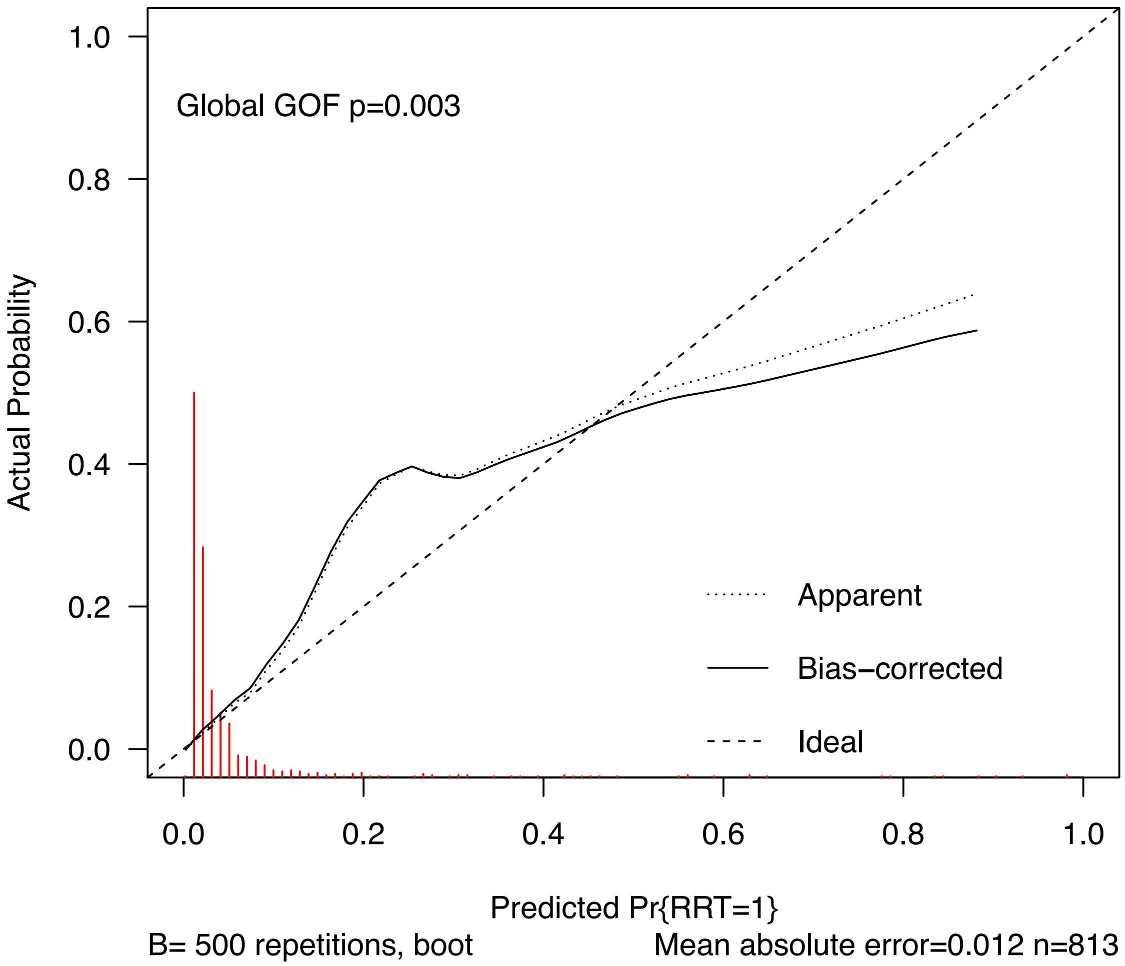

Fig S7: Calibration plot for RRT model

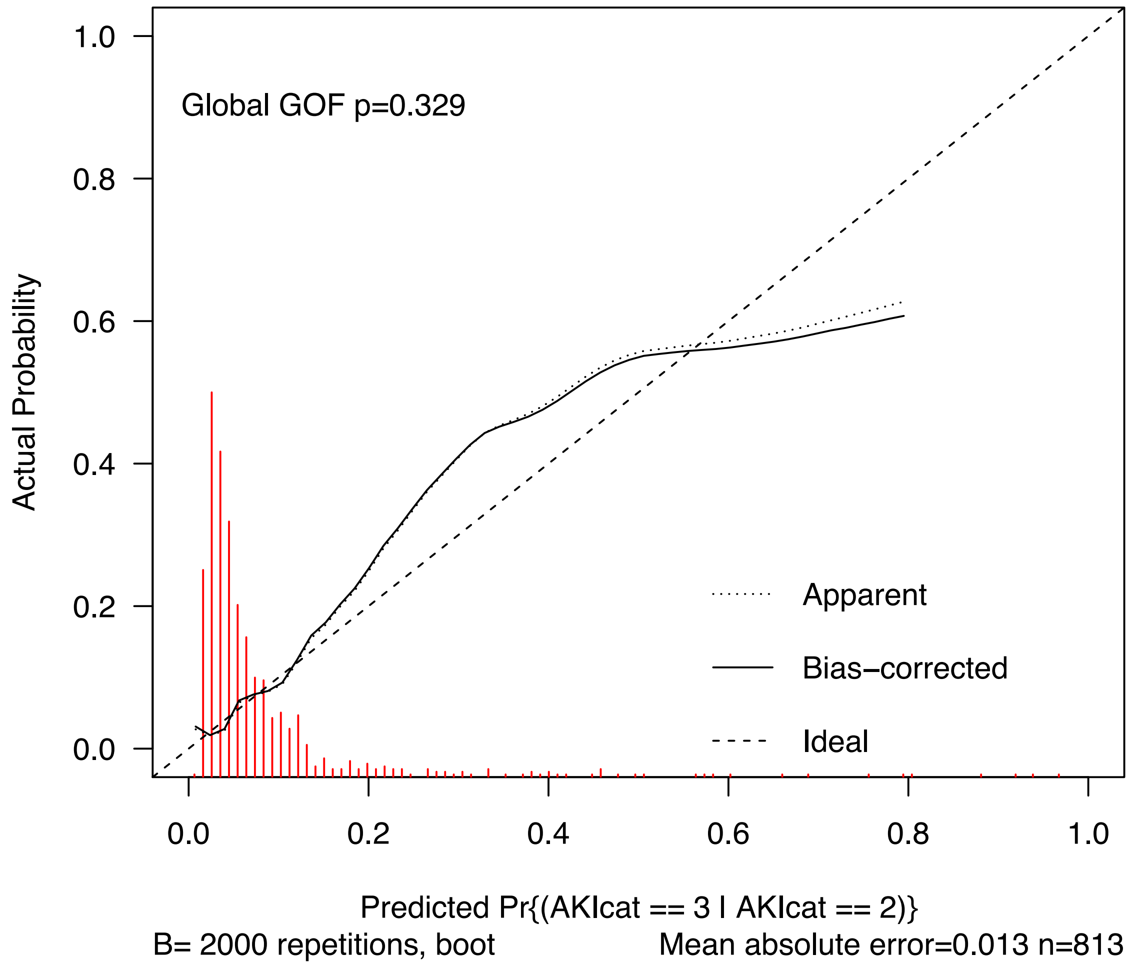

Fig S8: Calibration plot for AKI 2-3 model

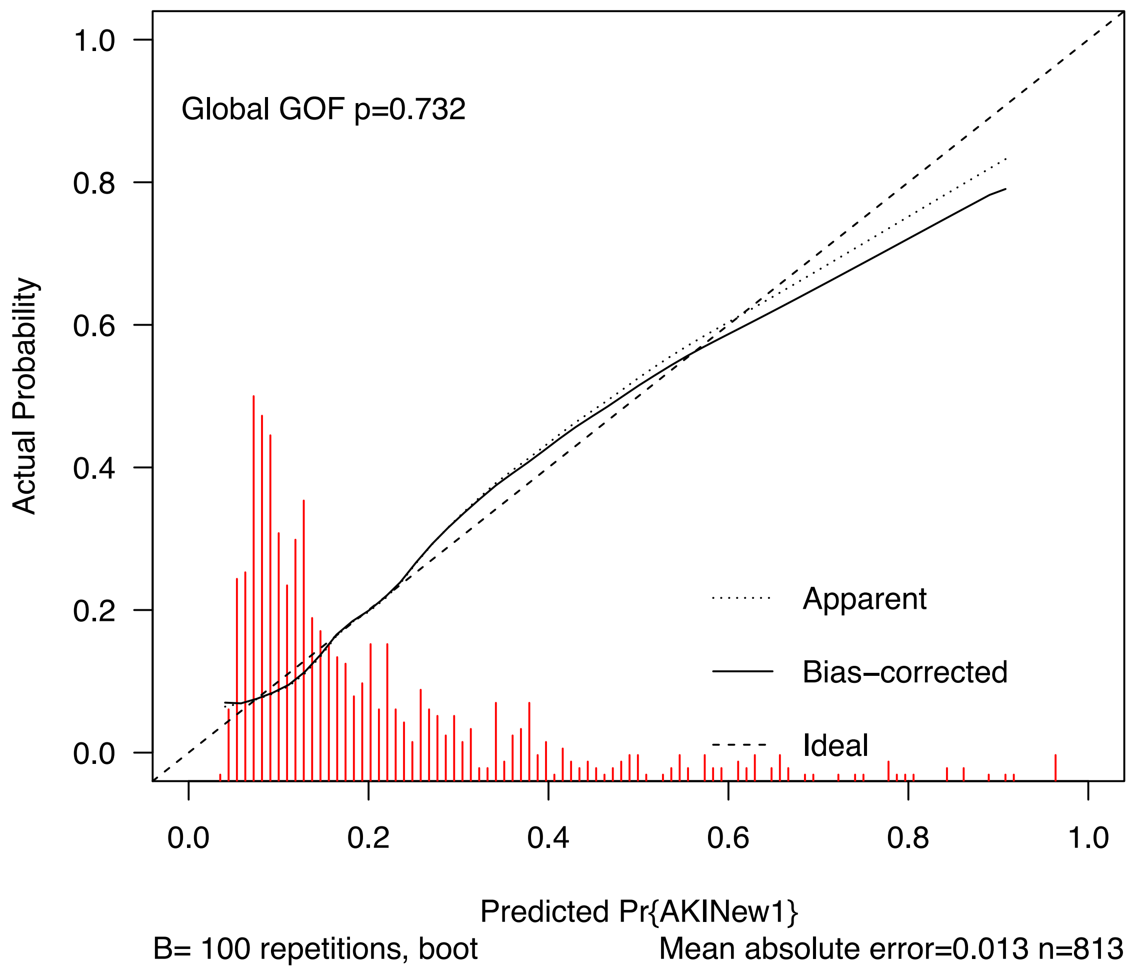

Fig S9: Calibration plot for Any AKI model

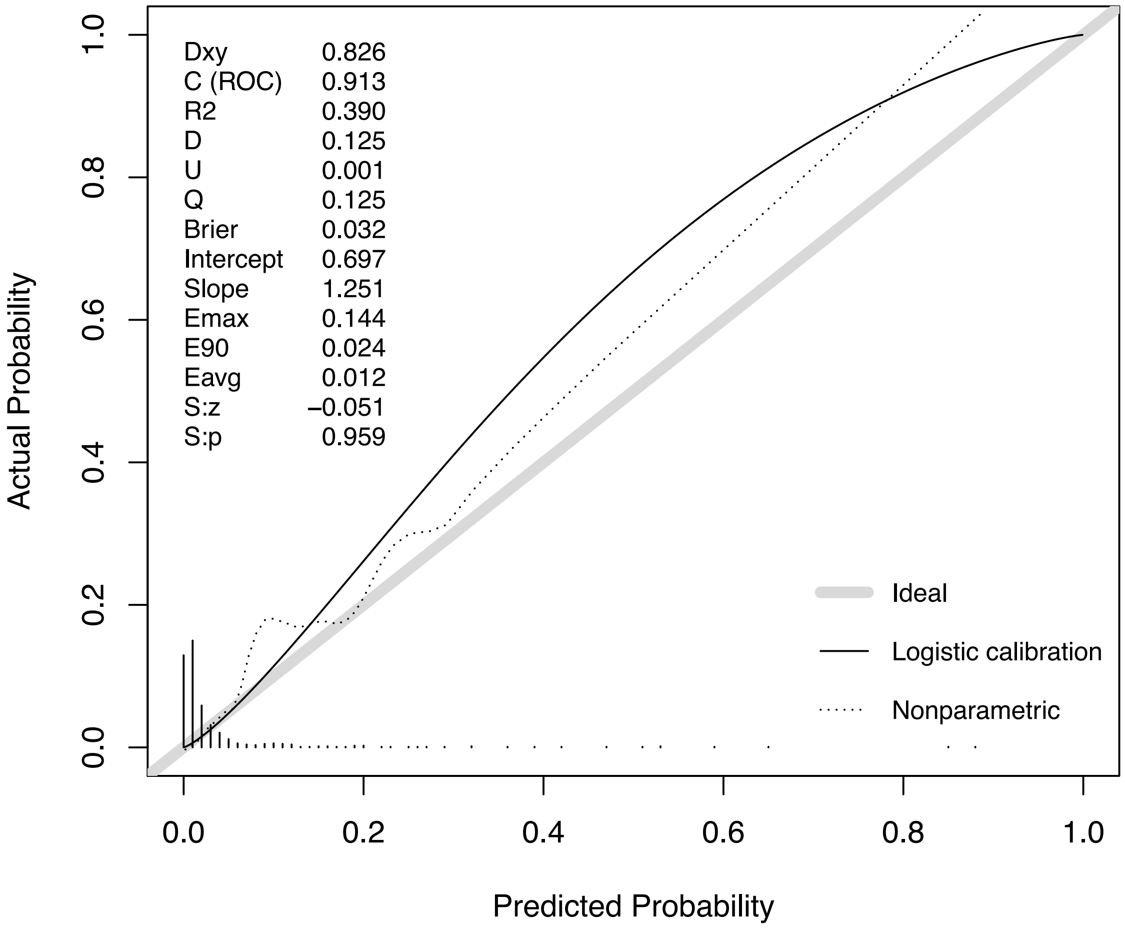

Fig S10: Calibration plot for RRT model – validation data

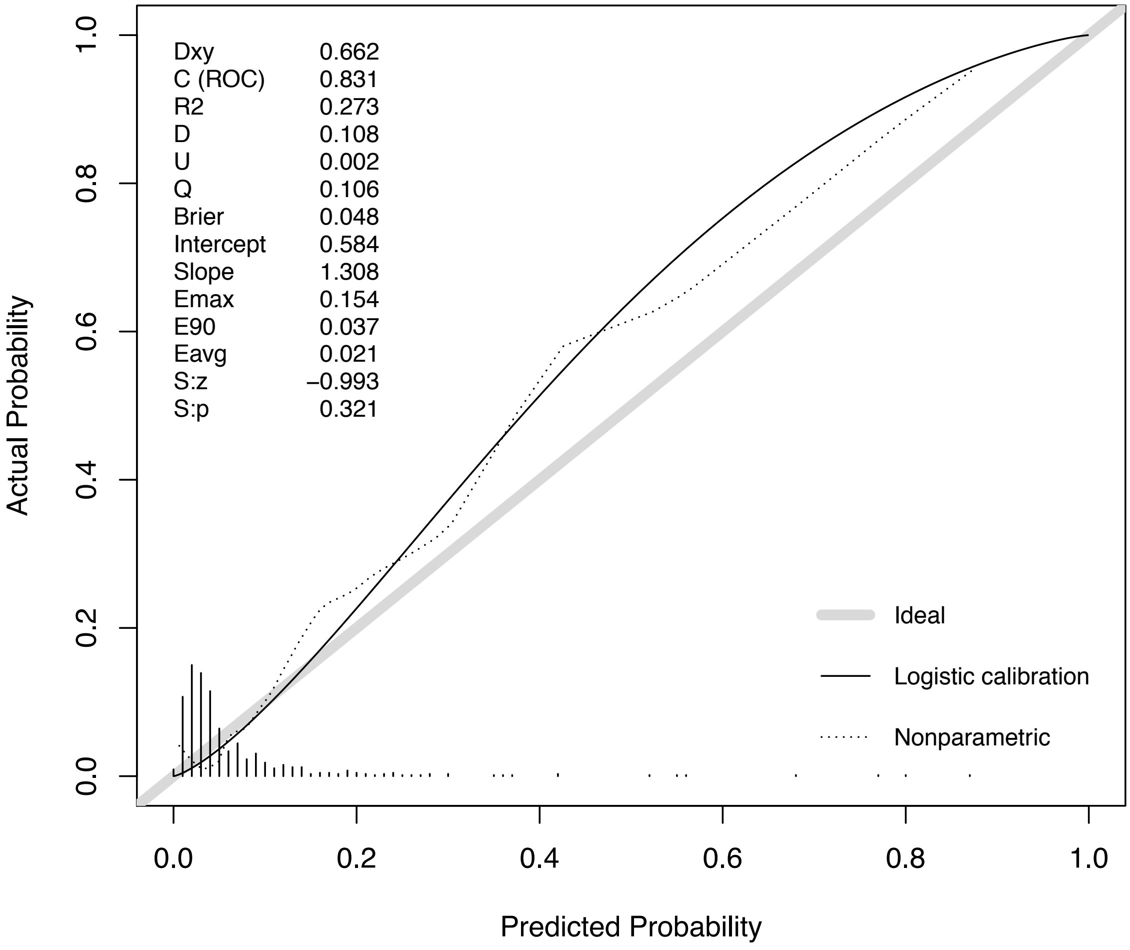

Fig S11: Calibration plot for AKI 2-3 model – validation data

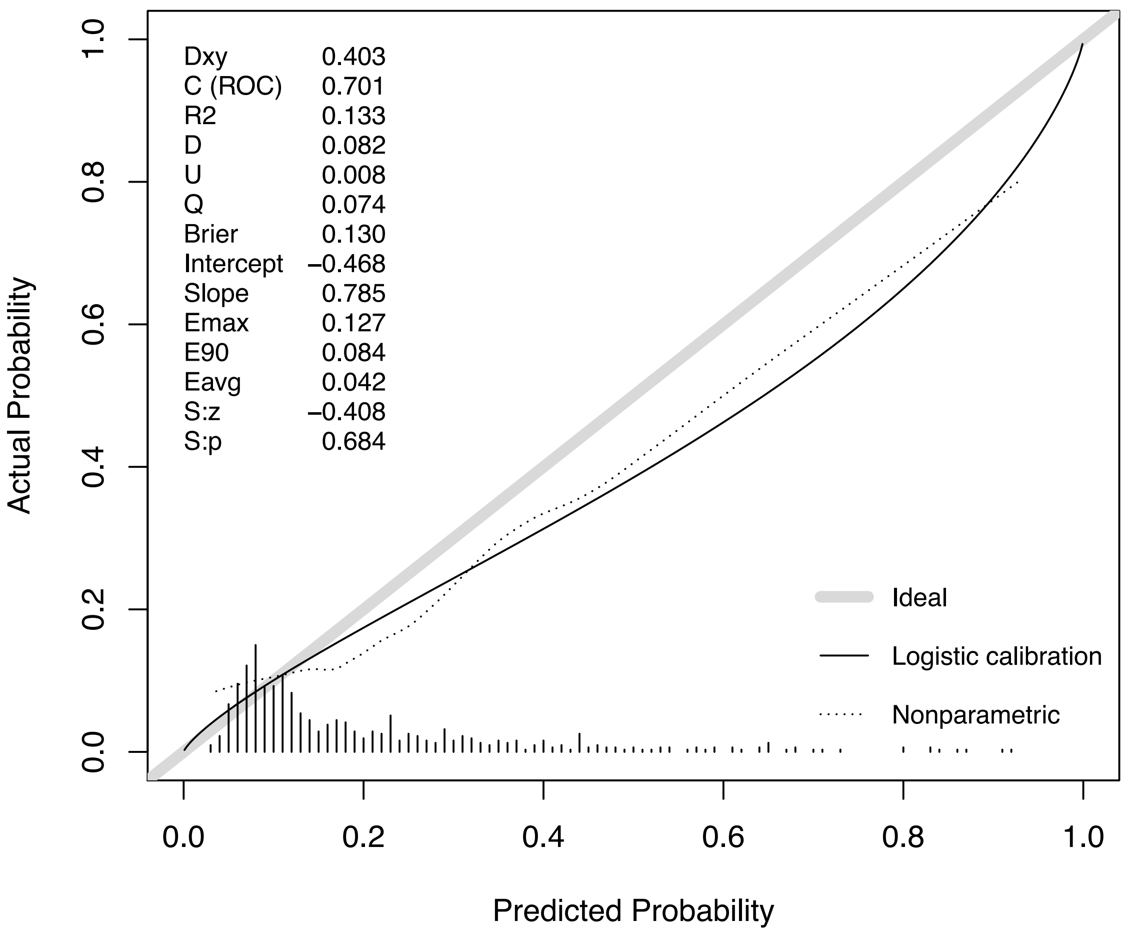

Fig S12: Calibration plot for Any AKI model – validation data

Classification and regression tree (CART) modelling

Finally, to explore differing cut-offs and the relative contribution of predictors in determining risk we examined the variables identified in logistic regression in classification and regression tree (CART) modelling in the entire patient cohort, using the R package *rpart*. The CART method involves the segregation of different values of classification variables through a decision tree composed of progressive binary splits and has been applied to risk prediction in critical illness. To avoid over-fitting, each tree was pruned to minimize the cross-validated error and risk of AKI was calculated for each of the terminal nodes. Pruned trees are presented below (Figs S13-15) and the performance of the cut-offs identified are presented in table (S3).

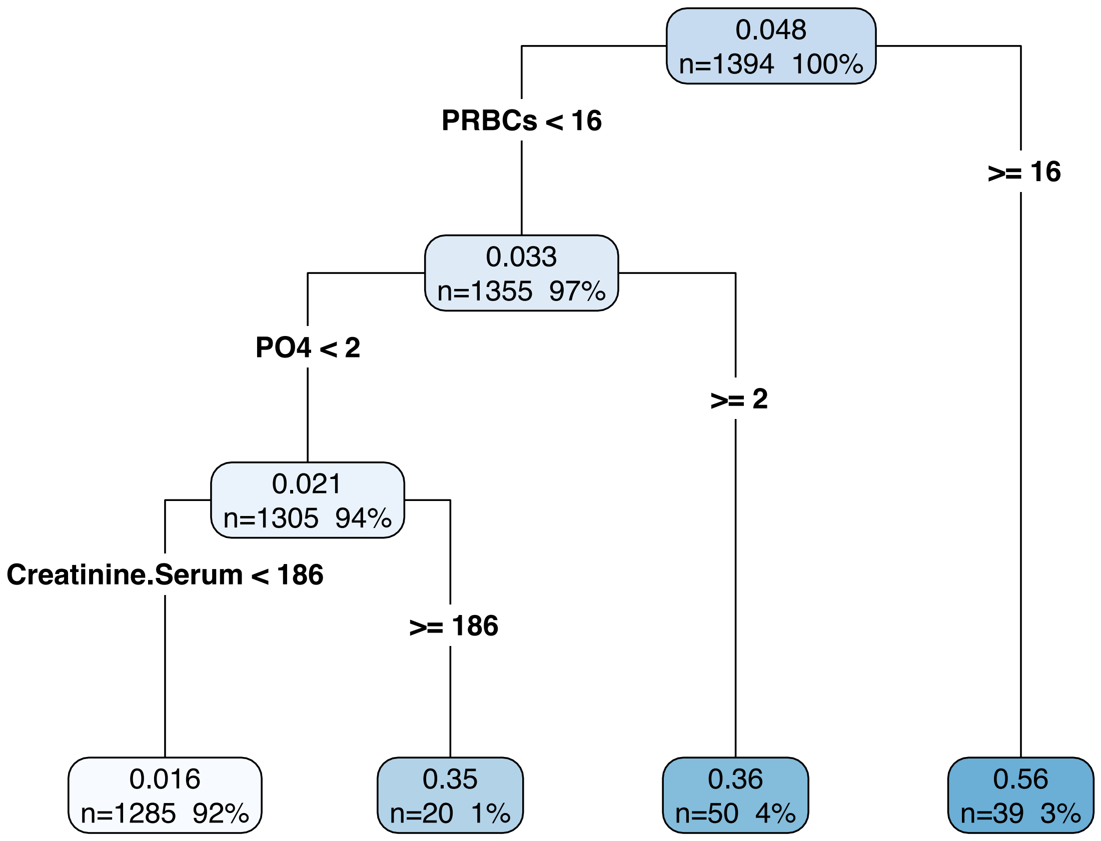

Fig S13: Decision tree for RRT. Terminal nodes used 2,3,4. PRBC - number of units of packed red cells transfused in first 24 h. PO4 – first serum phosphate.

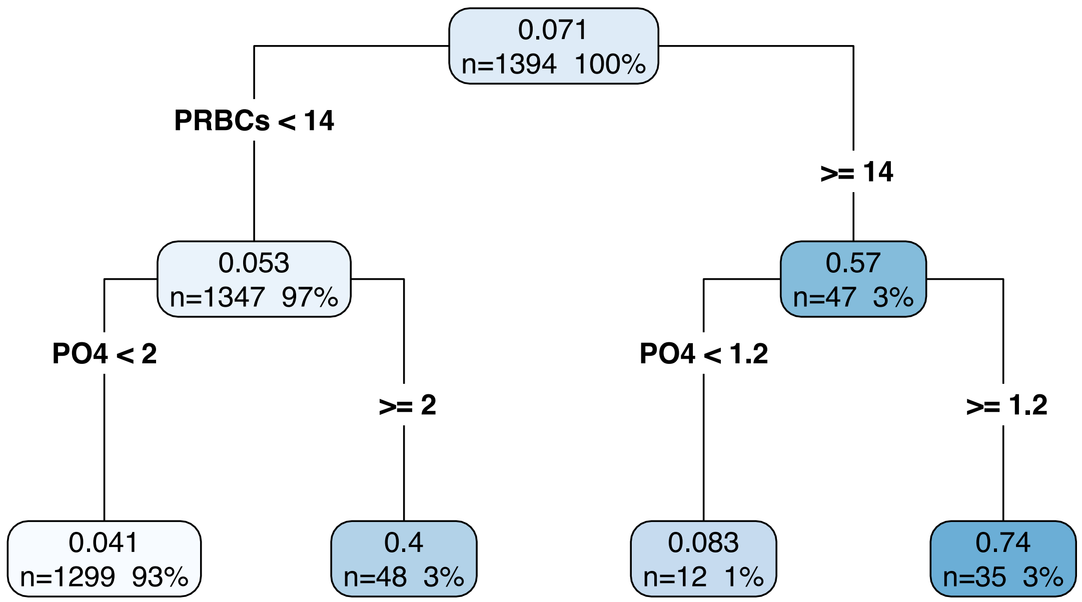

Fig S14: Decision tree for AKI 2-3. Terminal nodes used 2 and 4. PRBC - number of units of packed red cells transfused in first 24 h. PO4 – first serum phosphate.

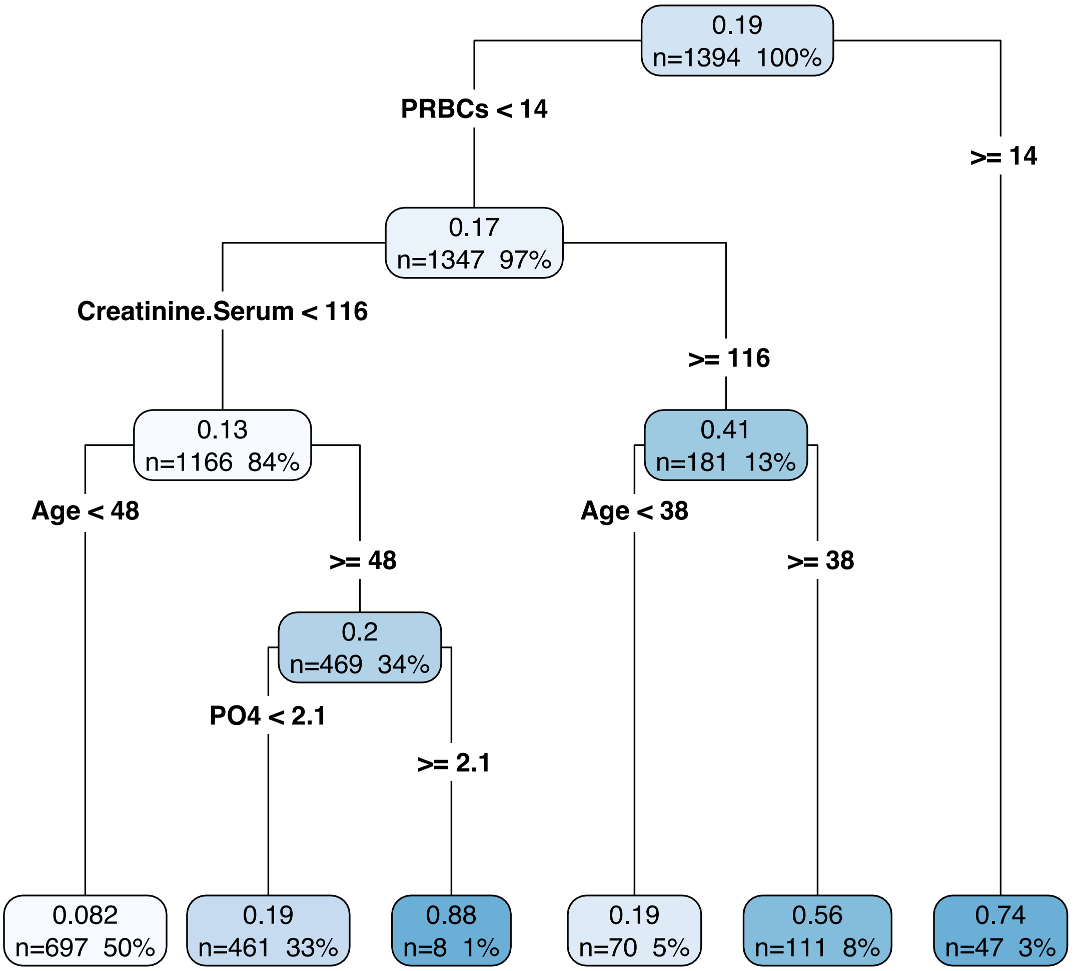

Fig S15: Decision tree for AKI 1-3. Terminal nodes used 3, 5 and 6. PRBC - number of units of packed red cells transfused in first 24 h. PO4 – first serum phosphate.
